# Discovering conservation laws using optimal transport and manifold learning

**DOI:** 10.1038/s41467-023-40325-7

**Published:** 2023-08-07

**Authors:** Peter Y. Lu, Rumen Dangovski, Marin Soljačić

**Affiliations:** 1 Data Science Institute, University of Chicago, Chicago, IL 60637 USA; 2 Department of Physics, Massachusetts Institute of Technology, Cambridge, MA 02139 USA; 3 Department of Electrical Engineering and Computer Science, Massachusetts Institute of Technology, Cambridge, MA 02139 USA

**Keywords:** Nonlinear phenomena, Computational science, Computer science, Statistics

## Abstract

Conservation laws are key theoretical and practical tools for understanding, characterizing, and modeling nonlinear dynamical systems. However, for many complex systems, the corresponding conserved quantities are difficult to identify, making it hard to analyze their dynamics and build stable predictive models. Current approaches for discovering conservation laws often depend on detailed dynamical information or rely on black box parametric deep learning methods. We instead reformulate this task as a manifold learning problem and propose a non-parametric approach for discovering conserved quantities. We test this new approach on a variety of physical systems and demonstrate that our method is able to both identify the number of conserved quantities and extract their values. Using tools from optimal transport theory and manifold learning, our proposed method provides a direct geometric approach to identifying conservation laws that is both robust and interpretable without requiring an explicit model of the system nor accurate time information.

## Abstract (web summary)

Conservation laws are crucial for analyzing and modeling nonlinear dynamical systems; however, identification of conserved quantities is often quite challenging. The authors propose here a geometric approach to discovering conservation laws directly from trajectory data that does not require an explicit dynamical model of the system or detailed time information.

## Introduction

Conservation laws are powerful constraints on the dynamics of many physical systems in nature, and the corresponding conserved quantities are essential features for characterizing the behavior of these systems. Through Noether’s theorem, conservation laws are closely tied with the symmetries of a physical system and play a key role in our understanding of physics. Conservation laws also help stabilize and enhance the performance of predictive models for complex nonlinear dynamics, e.g. symplectic integrators for Hamiltonian systems^1^ and pressure projection for incompressible fluid flow^2^. In fact, for chaotic dynamical systems, conserved quantities are often the only features of the system state that can be reliably known far into the future. Discovering conservation laws helps us characterize the long-term behavior of complex dynamical systems and understand the underlying physics.

While the conservation laws of many physical systems are well-known and often derived from known symmetries, there are still many instances where it is difficult to even determine the number of conservation laws, let alone explicitly extract the conserved quantities. As a historical example, consider the Korteweg–De Vries (KdV) equation modeling shallow water waves. The KdV equation, despite its apparent complexity, has infinitely many conserved quantities^3^ and is, in fact, fully solvable via an inverse scattering transform^4^—a discovery made after significant theoretical and computational effort. Developing better general methods for identifying conserved quantities will allow us to improve our understanding of new or understudied physical systems and build more efficient and stable predictive models.

In real-world applications, an accurate model for the underlying physical system is often unavailable, forcing us to identify conservation laws using only sample trajectories of the system dynamics. One broad approach is to use modern data-driven methods based on the Koopman operator formulation of dynamical systems, which lifts the dynamics into an infinite dimensional operator space^5^. In the Koopman formalism, conserved quantities are just one type of Koopman eigenfunction with eigenvalue zero. Thus, one approach is to first apply a system identification method, such as dynamic mode decomposition^6,7^, sparse identification with a library of basis functions^8^, or even deep learning-based approaches^9–11^, to model the system dynamics and then set up and solve the Koopman eigenvalue problem. Alternatively, previous work has also proposed directly setting up the eigenvalue problem by estimating time derivatives from data and then fitting the conserved quantities using a library of possible terms^12^ or a neural network^13^. These methods can work quite well but require that the measured trajectories have sufficiently low noise and high time resolution in order to accurately estimate time derivatives.

Constructing a model for a dynamical system provides much more information than just the conservation laws. In fact, even estimating time derivatives is usually not necessary if we are only interested in identifying conserved quantities. In this work, we will instead focus on an alternative approach that does not require an explicit model or detailed time information but rather takes advantage of the geometric constraints imposed by conservation laws. Specifically, the presence of conservation laws restricts each trajectory in phase space to lie solely on a lower dimensional isosurface of the conserved quantities. The dimensionality of these isosurfaces can provide information about the number of conserved quantities or constraints^14^. Furthermore, since each isosurface corresponds to a particular set of conserved quantities, the variations in shape of the isosurfaces directly correspond to variations in the conserved quantities. In other words, we can identify and extract conserved quantities by examining the varying shapes of the isosurfaces sampled by the trajectories.

**Fig. 1:**
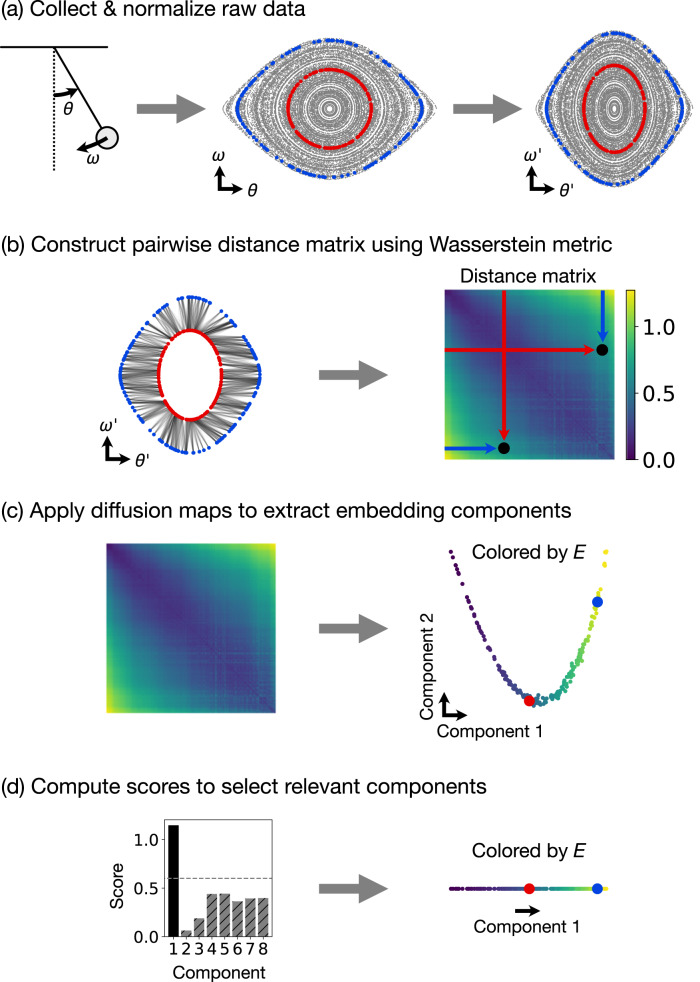
Proposed non-parametric method for discovering conservation laws illustrated using a simple pendulum example. **a** First, we collect and normalize the trajectory data from the dynamical system. Two example trajectories are highlighted in red and blue. **b** Then, we use the Wasserstein metric from optimal transport to compute the distance between each pair of trajectories and construct a distance matrix. For the two example trajectories, the optimal transport plan is shown as lines connecting pairs of points. The constructed distance matrix is plotted with color representing the computed Wasserstein distance between each pair of trajectories. The computed distance between the two example trajectories is marked (black dots) on the distance matrix plot. **c** An embedding of the shape space manifold ${{{{{{{\mathcal{C}}}}}}}}$ is extracted from the distance matrix using diffusion maps. The embedding plot is colored by the conserved energy of the pendulum *E*. The points corresponding to the two example trajectories are marked in red and blue. **d** Finally, a heuristic score (Supplementary Note 1) is used to select relevant components. In this case, only component 1 is relevant, corresponding to a single conserved quantity—the energy *E*. Again, the embedding plot is colored by *E*, and the two example trajectories are marked in red and blue.

In contrast with recent work using black box deep learning methods to fit conserved quantities that are consistent with the sampled isosurfaces^15,16^, we propose and demonstrate a non-parametric manifold learning approach (Fig. 1) that directly characterizes the variations in the sampled isosurfaces, producing an embedding of the space of conserved quantities. Our method first uses the Wasserstein metric from optimal transport^17^ to compute distances in shape space between pairs of sampled isosurfaces and then extracts a low dimensional embedding for the manifold of isosurfaces using diffusion maps^18,19^. Each point in this embedding corresponds to a distinct isosurface and therefore to a distinct set of conserved quantities, i.e. the embedding explicitly parameterizes the space of varying conserved quantities. Related methods have been recently suggested for characterizing molecular conformations using the 1-Wasserstein distance together with diffusion maps^20^, performing system identification by comparing invariant measures using the 2-Wasserstein distance^21^, and reconstructing normal forms using diffusion maps^22^. Recent theoretical work has also formalized the idea of using alternative non-Euclidean norms, like the Wasserstein distance, in spectral embedding methods such as diffusion maps^23^.

We provide an analytic analysis of our approach for a simple harmonic oscillator system and numerically test our method on several physical systems: the single and double pendulum, planar gravitational dynamics, the KdV equation for shallow water waves, and a nonlinear reaction–diffusion equation that generates an oscillating Turing pattern. We also demonstrate the robustness of our approach to noise in the measured trajectories, missing information in the form of a partially observed phase space, as well as approximate conservation laws (additional experiments in Supplementary Notes 3 and 5). In our comparison tests (Supplementary Note 6), our approach outperforms prior deep learning-based direct fitting methods, all while being an order of magnitude faster. We also provide an easy-to-use codebase (https://github.com/peterparity/conservation-laws-manifold-learning), which parallelizes across multiple GPUs, to make an efficient implementation of our method as accessible as possible.

## Results

### Analytic result for the simple harmonic oscillator

In the case of a simple harmonic oscillator (SHO) without measurement noise and in the infinite sample limit, we are able to explicitly derive an analytic result for our proposed procedure. We first compute the pairwise distances provided by the Wasserstein metric and then derive the embedding produced by a diffusion map, which corresponds to the conserved energy of the SHO.

#### Wasserstein metric: constructing the isosurface shape space

Consider a SHO with Hamiltonian

1 $$H(q,\,p)=\frac{1}{2m}{p}^{2}+\frac{1}{2}m{\omega }^{2}{q}^{2}$$

given in terms of position *q* and momentum *p*. The SHO energy isosurfaces *E* = *H*(*q*, *p*) form concentric ellipses in a 2D phase space. Choosing units such that *m* = 1 and *ω* = 1, we obtain concentric circles with uniformly distributed samples (assuming a uniform sampling in time). The 2-Wasserstein distance between a pair of uniformly distributed circular isosurfaces is simply given by the difference in radii $\left|{r}_{1}-{r}_{2}\right|$. This is because, due to the rotational symmetry of the two distributions, the optimal transport plan for an isotropic cost function is to simply move each point on isosurface 1 radially outward (or inward) to the point on isosurface 2 with the same angle *θ*.

This result does not meaningfully change with a different choice of units, which is equivalent to rescaling the phase space coordinates *q*, *p*. If we rescale *q*, *p* by factors *k*_*q*_, *k*_*p*_, our cost function simply becomes

2 $$c({\theta }_{i},\,{\theta }_{j})={k}_{q}^{2}{({r}_{1}\cos {\theta }_{i}-{r}_{2}\cos {\theta }_{j})}^{2}+{k}_{p}^{2}{({r}_{1}\sin {\theta }_{i}-{r}_{2}\sin {\theta }_{j})}^{2},$$

where we label points on the isosurfaces by their angle *θ* on the original circular isosurfaces. The SHO optimal transport plan Π takes *θ* on isosurface 1 to the point with the same angle *θ* on isosurface 2, and Π for the SHO is invariant to coordinate rescaling (Supplementary Note 9). Therefore, the total transport cost is

3 $$C=\frac{1}{2\pi }\int\nolimits_{0}^{2\pi }c(\theta,\,\theta )\,{{{{{{{\rm{d}}}}}}}}\theta=\frac{{k}_{q}^{2}+{k}_{p}^{2}}{2}{({r}_{1}-{r}_{2})}^{2},$$

so the 2-Wasserstein distance is

4 $$\sqrt{C}=\sqrt{({k}_{q}^{2}+{k}_{p}^{2})/2}\,\left|{r}_{1}-{r}_{2}\right|\propto \left|{r}_{1}-{r}_{2}\right|,$$

i.e. the same result modulo a constant factor. While this is not a general result, we find that our approach is often fairly robust to such changes, including the extreme case of scaling some phase space coordinates all the way down to zero resulting in a partially observed phase space (Supplementary Note 5).

#### Diffusion maps: extracting the conserved energy

Once we have pairwise distances in the isosurface shape space, we can use diffusion maps to study the resulting manifold of isosurface shapes. With sufficient samples, the operator constructed by the diffusion map should converge to the Laplace–Beltrami operator on the manifold. For the SHO, the isosurface shape space is isomorphic to ${{\mathbb{R}}}^{+}$ with each circular isosurface mapped to its radius. If we sample trajectories with radii $r\in (0,\sqrt{2{E}_{0}})$ for some maximum energy *E*_0_, then the manifold is a real line segment, and the resulting Laplacian operator (with open boundary conditions) has eigenvalues *λ*_*n*_ = *π*^2^*n*^2^/2*E*_0_ and corresponding eigenvectors ${v}_{n}(r)=\cos (\sqrt{{\lambda }_{n}}\,r)$. Therefore, the first eigenvector or embedding component

5 $${v}_{1}(E)=\cos (\pi \sqrt{E/{E}_{0}})$$

successfully encodes the conserved energy and is, in fact, a monotonic function of the energy.

### Numerical experiments

To demonstrate and empirically test our method for discovering conservation laws, we generate datasets from a wide range of dynamical systems, each consisting of randomly sampled trajectories with different initial conditions and the corresponding conserved quantities. Note that we use the dimensionless form of each dynamical system. All of the code necessary for reproducing our results is available at https://github.com/peterparity/conservation-laws-manifold-learning.

#### Simple Harmonic Oscillator

**Fig. 2:**
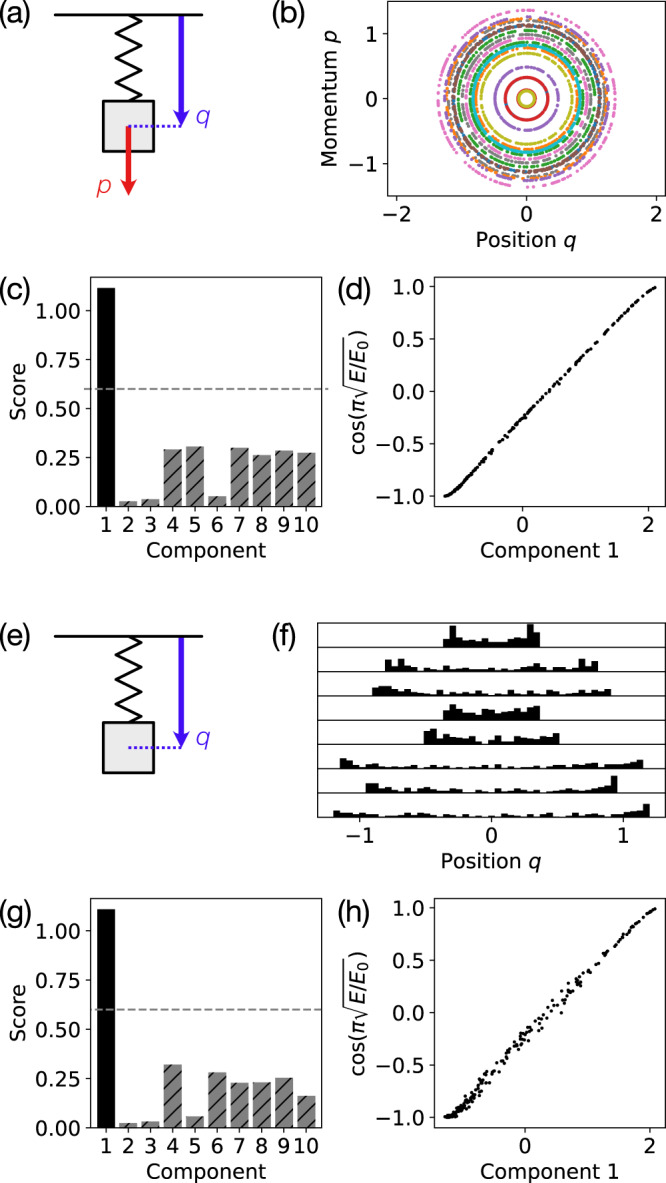
Identifying the conserved energy for the simple harmonic oscillator (SHO). **a** The SHO has two degrees of freedom: position *q* and momentum *p*. **b** Sample trajectories from the SHO dataset show sample points plotted in the 2D phase space (*q*, *p*). **c** The heuristic score (with cutoff 0.6) correctly identifies that the first embedding component extracted by the diffusion map is the only relevant component. **d** The extracted first component closely matches the analytically predicted first component (Eq. (5)) for the SHO (*R*^2^ = 0.9995). **e** Next, consider the SHO dataset with a partially observed phase space containing position only. **f** For each sample trajectory, the sample points are shown as a histogram. **g** The heuristic score is still able to identify the first component as relevant, and **h** this first component matches the analytic prediction (*R*^2^ = 0.9961).

We first numerically test our analytic result for the SHO and obtain good agreement (Fig. 2) using both the default scaling *k*_*q*_ = *k*_*p*_ = 1 (Fig. 2a–d) as well as the position only scaling *k*_*q*_ = 1, *k*_*p*_ = 0 (Fig. 2e–h), which effectively reduces the dimension of the phase space. A linear fit of the first embedding component from the diffusion map with the analytically predicted component (Eq. (5)) achieves a correlation coefficient of *R*^2^ = 0.9995 for the default scaling and *R*^2^ = 0.9961 for the position only scaling. We also verify that the heuristic score (Supplementary Note 1) accurately determines that there is only one relevant embedding component (Fig. 2c, g), which corresponds to the conserved energy.

#### Simple pendulum

**Fig. 3:**
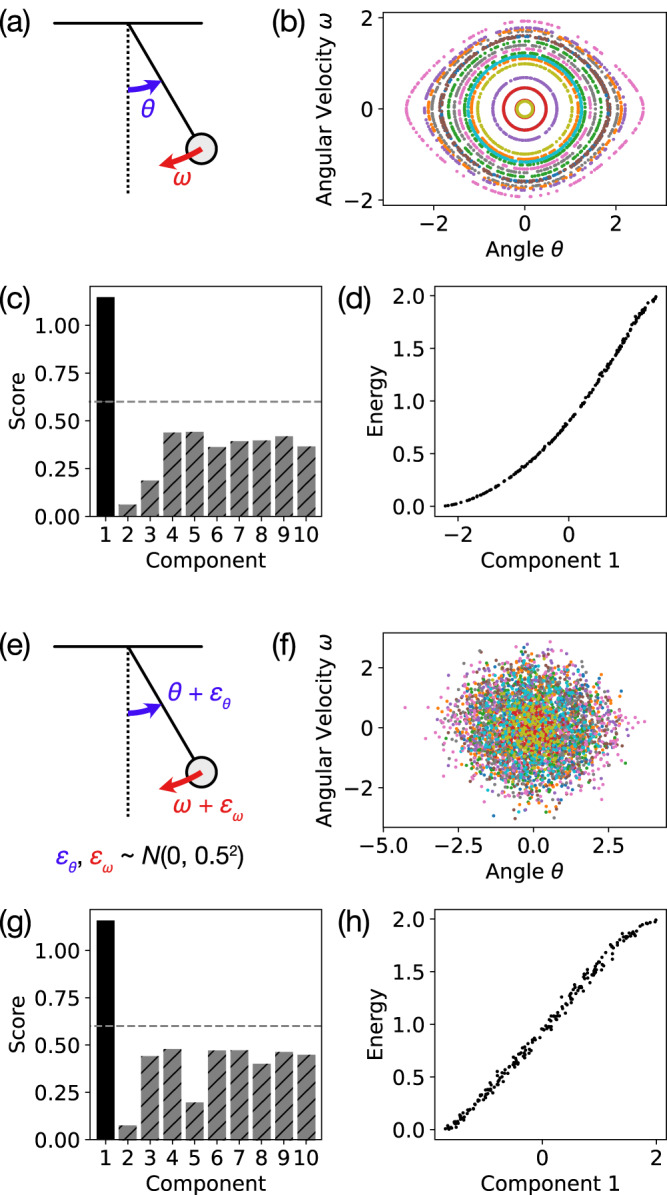
Identifying the conserved energy for the simple pendulum. **a** The simple pendulum has two degrees of freedom: angular position *θ* and angular velocity *ω*. **b** Sample trajectories show sample points plotted in the 2D phase space (*θ*, *ω*). **c** The heuristic score (with cutoff 0.6) correctly identifies that the first embedding component extracted by the diffusion map is the only relevant component, and **d** the extracted first component is monotonically related to the energy (rank correlation *ρ* = 0.9997). **e**, **f** With the addition of *σ* = 0.5 Gaussian noise to simulate measurement noise, **g** the heuristic score is still able to identify the first component as relevant, and (h) this first component corresponds well to the energy (*ρ* = 0.9978).

To demonstrate our method on a simple nonlinear dynamical system, we analyze a simple pendulum that has a 2D phase space consisting of the angle *θ* and angular momentum *ω* (Fig. 3a). The equations of motion are

6 $$\frac{{{{{{{{\rm{d}}}}}}}}\omega }{{{{{{{{\rm{d}}}}}}}}t}=	-\sin \theta \\ \frac{{{{{{{{\rm{d}}}}}}}}\theta }{{{{{{{{\rm{d}}}}}}}}t}=	\omega.$$

This system has a single scalar conserved quantity

7 $$E=\frac{1}{2}{\omega }^{2}+(1-\cos \theta )$$

corresponding to the total energy of the pendulum, so the trajectories form 1D orbits in phase space (Fig. 3b).

Our method is able to correctly determine that there is only a single conserved quantity (Fig. 3c) corresponding to the energy of the pendulum (Fig. 3d). The single extracted embedding component is monotonically related to the energy with Spearman’s rank correlation coefficient *ρ* = 0.9997. We are also able to achieve similar results (*ρ* = 0.9978) with a high level of Gaussian noise (standard deviation *σ* = 0.5) added to the raw trajectory data (Fig. 3e–h), showing that our approach is quite robust to measurement noise.

#### Planar gravitational dynamics

**Fig. 4:**
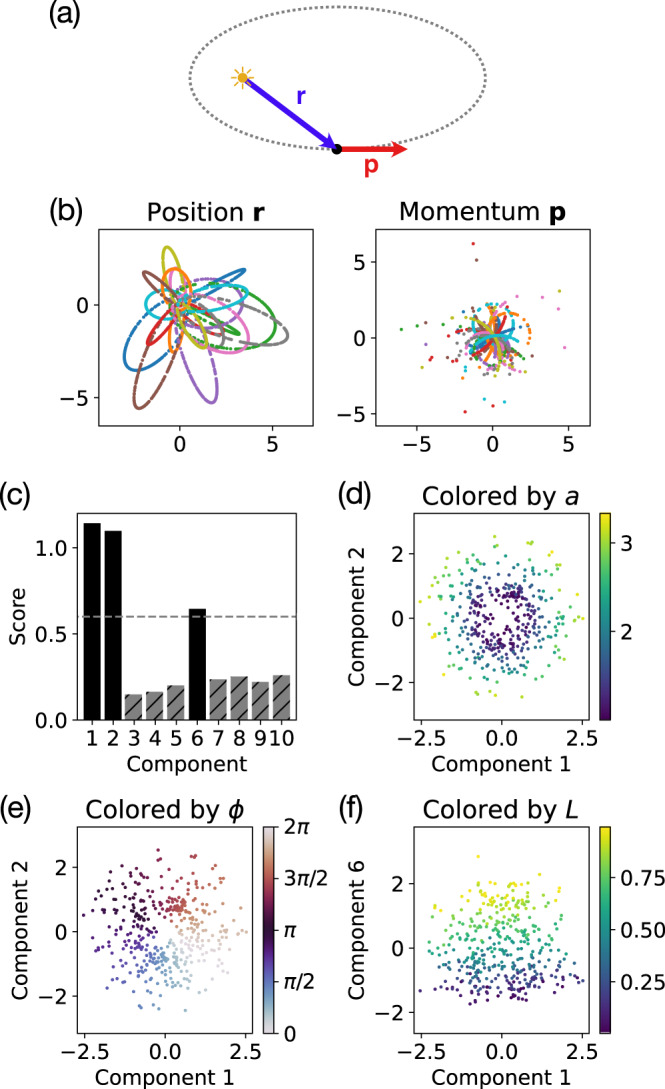
Identifying conserved quantities for planar gravitational dynamics. **a** Planar gravitational dynamics has four degrees of freedom: position vector **r** and momentum vector **p**. **b** Sample trajectories show sample points plotted in 2D slices of the 4D phase space consisting of position **r** and momentum **p**. **c** The heuristic score (with cutoff 0.6) identifies three relevant embedding components corresponding to the three independent conserved quantities. **d**, **e** Components 1 and 2 embed the semi-major axis vector **a** with magnitude *a* = − 1/2*E* related to the energy and orientation given by the angle *ϕ*. **f** Component 6 corresponds to the angular momentum *L*.

To test our method on a system with multiple conserved quantities, we simulate the gravitational system of a planet orbiting a star with much greater mass (Fig. 4a). We fix the orbits to all lie in a 2D plane, giving us an effectively 4D phase space. The resulting equations of motion are

8 $$\frac{{{{{{{{\rm{d}}}}}}}}{{{{{{{\bf{r}}}}}}}}}{{{{{{{{\rm{d}}}}}}}}t}=	{{{{{{{\bf{p}}}}}}}}\\ \frac{{{{{{{{\rm{d}}}}}}}}{{{{{{{\bf{p}}}}}}}}}{{{{{{{{\rm{d}}}}}}}}t}=	-\frac{{{{{{{{\hat{{{\bf{r}}}}}}}}}}}}{{\left|{{{{{{{\bf{r}}}}}}}}\right|}^{2}}.$$

This system has one scalar and two vector-conserved quantities

9 $$E=	\frac{{{{{{{{{\bf{p}}}}}}}}}^{2}}{2}-\frac{1}{\left|{{{{{{{\bf{r}}}}}}}}\right|}\\ {{{{{{{\bf{L}}}}}}}}=	{{{{{{{\bf{r}}}}}}}}\times {{{{{{{\bf{p}}}}}}}}\\ {{{{{{{\bf{A}}}}}}}}=	{{{{{{{\bf{p}}}}}}}}\times {{{{{{{\bf{L}}}}}}}}-{{{{{{{\hat{{{\bf{r}}}}}}}}}}},$$

which, in our 4D phase space, reduces to three scalar conserved quantities: the total energy *E* (or equivalently, the semi-major axis *a* = − 1/2*E*), the angular momentum $L=\left|{{{{{{{\bf{L}}}}}}}}\right|$, and the orbital orientation angle *ϕ*, which is the angle of the LRL vector **A** relative to the *x*-axis. As a result, the trajectories also form 1D orbits in the phase space (Fig. 4b).

Our approach accurately identifies the three conserved quantities (Fig. 4c), and the extracted embedding corresponds most directly to the geometric features of the orbits (Fig. 4d–f). The first two components embed the semi-major axis vector ${{{{{{{\bf{a}}}}}}}}=(a\cos \phi,a\sin \phi )$ with magnitude given by the semi-major axis *a* = − 1/2*E*, which is related to the energy *E*, and orientation given by the orientation angle *ϕ* of the elliptical orbit (Fig. 4d, e). The third relevant component (component 6) embeds the angular momentum *L* (Fig. 4f). See Supplementary Note 1.1 for details on choosing a cutoff to identify the relevant components. A linear fit of the identified relevant embedding components with $a\cos \phi$ ($a\sin \phi$) has *R*^2^ = 0.987 (*R*^2^ = 0.986) and rank correlation *ρ* = 0.994 (*ρ* = 0.992). A similar linear fit with *L* has *R*^2^ = 0.927 and *ρ* = 0.970.

This example demonstrates that, for a system with multiple conserved quantities, the ground metric for optimal transport controls the relative scale of each conserved quantity in the extracted embedding. In this case, the geometry of the shape space ${{{{{{{\mathcal{C}}}}}}}}$ is dominated by changes in the semi-major axis *a* and orientation angle *ϕ*, whereas changes in the angular momentum *L*, which controls the eccentricity of the orbit, play a more minor role and thus appear in a later embedding component with a lower score (Fig. 4c).

#### Double pendulum

**Fig. 5:**
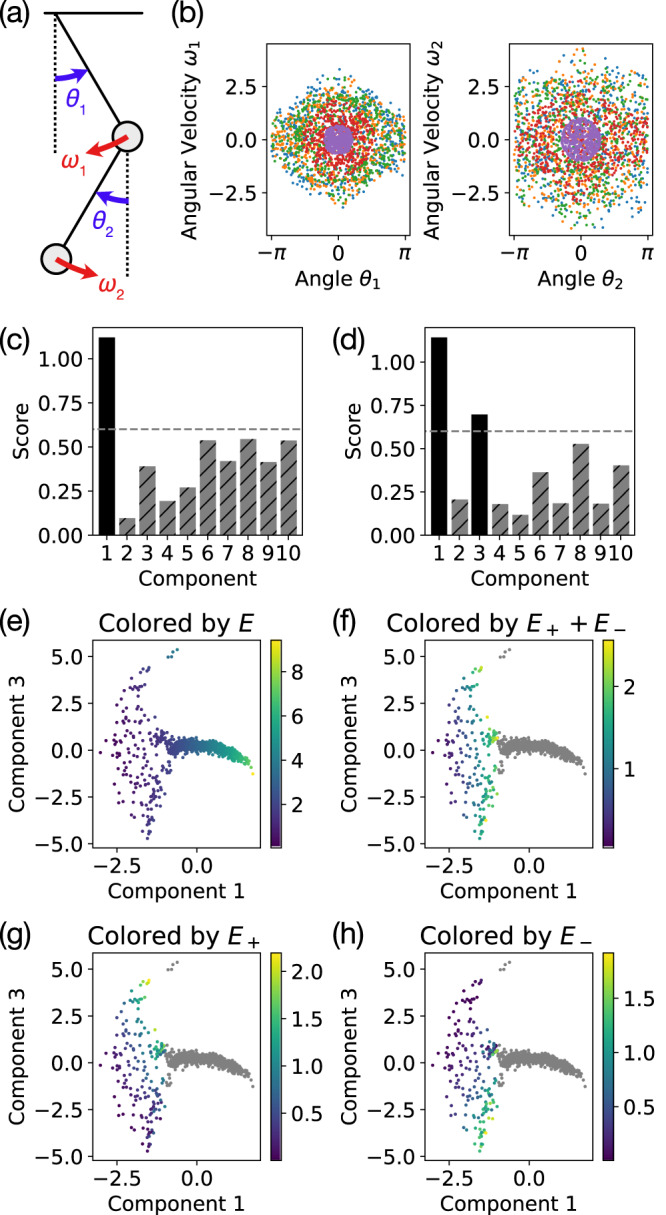
Identifying conserved quantities for the double pendulum. **a** The double pendulum has four degrees of freedom: angular positions *θ*_1_, *θ*_2_ and angular velocities *ω*_1_, *ω*_2_. **b** Sample trajectories show sample points plotted in 2D slices of the 4D phase space. **c** The heuristic score (with cutoff 0.6) identifies one relevant embedding component corresponding to (**e**) the total energy *E*. **d** However, if we restrict the embedding to trajectories with first component *v*_1_ < − 1 (i.e. low energy trajectories) and renormalize the embedding, **f–h** we find two conserved quantities corresponding to the energies *E*_±_ of the two decoupled low energy modes. The gray points in (**f**–**h**) correspond to the high energy trajectories (first component *v*_1_ > − 1) which are not relevant when considering the low-energy non-chaotic phase of the double pendulum.

To test our approach on a non-integrable system with higher dimensional isosurfaces, we study the classic double pendulum system (Fig. 5a) with unit masses and unit-length pendulum arms. This system has a 4D phase space, consisting of the angles *θ*_1_, *θ*_2_ and the angular velocities *ω*_1_, *ω*_2_ of the two pendulums (Fig. 5b), and only has a single scalar conserved quantity

10 $$E={\omega }_{1}^{2}+\frac{1}{2}{\omega }_{2}^{2}+{\omega }_{1}{\omega }_{2}\cos ({\theta }_{1}-{\theta }_{2})-2\cos {\theta }_{1}-\cos {\theta }_{2}$$

corresponding to the total energy. However, the double pendulum system has both chaotic and non-chaotic phases. In particular, at high energies, the system is chaotic and only conserves the total energy, while at low energies, the system behaves more like two coupled harmonic oscillators with two independent (approximately) conserved energies

11 $${E}_{\pm }=\frac{1}{8}\left[4{\theta }_{1}^{2}+2{\theta }_{2}^{2}\pm \sqrt{2}\,{\theta }_{1}{\theta }_{2}+\left(2\pm \sqrt{2}\right)\left(2{\omega }_{1}^{2}+{\omega }_{2}^{2}\right)+4\left(1\pm \sqrt{2}\right){\omega }_{1}{\omega }_{2}\right]$$

corresponding to the two modes of the coupled oscillator system. Therefore, we expect to see two distinct phases in our extracted embedding: one with a single conserved quantity *E* at high energy and another with two approximately conserved quantities *E*_±_ at low energy, which approximately sum to *E* ≈ *E*_+_ + *E*_−_.

At first glance, it appears as though our method has only identified a single relevant component corresponding to the conserved total energy *E* (Fig. 5c, e) with rank correlation *ρ* = 0.996. However, if we restrict ourselves to low-energy trajectories with first embedding component *v*_1_ < − 1, we find that there is a region of the shape space that is two-dimensional, corresponding to the two independently conserved energies *E*_±_ of the low-energy non-chaotic phase where the double pendulum behaves like a coupled oscillator system with two distinct modes. For the low energy trajectories, a linear fit of the now two relevant components with *E*_+_ (*E*_−_) has rank correlation *ρ* = 0.919 (*ρ* = 0.937). If we restrict ourselves to even lower energy trajectories with *v*_1_ < − 2, a similar linear fit for *E*_+_ (*E*_−_) has rank correlation *ρ* = 0.990 (*ρ* = 0.989).

This analysis of the double pendulum shows that our method can still provide significant insight into complex dynamical systems with multiple phases involving varying numbers of conserved quantities. This manifests itself as manifolds of different dimensions in shape space that are stitched together at phase transitions, presenting a significant challenge for most manifold learning methods. In this example, this difficulty is reflected in the performance of the heuristic score (Fig. 5c,d), which has trouble telling whether the embedding is one or two-dimensional precisely because it is a combination of both a one and two-dimensional manifold. The embedding, on the other hand, remains very informative despite the sudden change in dimensionality and allows us to identify interesting features of the system, such as nonlinear periodic orbits (see Supplementary Note 4). The effectiveness of diffusion maps when handling these complex situations has been previously observed in parameter reduction applications^24^ and is worth studying in more detail in the future.

#### Oscillating turing patterns

Next, we consider an oscillating Turing pattern system that is both dissipative and has a much higher dimensional phase space than our previous examples. In particular, we study the Barrio–Varea–Aragón–Maini (BVAM) model^25,26^

12 $$\frac{\partial u}{\partial t}=D\frac{{\partial }^{2}u}{\partial {x}^{2}}+u-v-Cuv-u{v}^{2}\\ \frac{\partial v}{\partial t}=\frac{{\partial }^{2}v}{\partial {x}^{2}}-\frac{3}{2}v+Hu+Cuv+u{v}^{2}$$

with *D* = 0.08, *C* = − 1.5, and *H* = 3, following Aragón et al.^26^ who showed that this set of parameters results in a spatial Turing pattern that also exhibits chaotic oscillating temporal dynamics, on a periodic domain with size 8. In our method, each trajectory [*u*(*x*, *t*_*i*_), *v*(*x*, *t*_*i*_)], *i* ∈ {1, 2, …, *N*} is treated as an unordered set of sample points in phase space, so we refer to the phase space as [*u*(*x*), *v*(*x*)] in a slight abuse of notation. The phase space of the BVAM system consists of two functions *u*(*x*) and *v*(*x*) which we discretize on a mesh of size 50, giving us an effective phase space dimension of 100. Because this system is dissipative, we will focus on characterizing the long-term behavior of the dynamics, i.e. the oscillating Turing pattern, which appears to have a conserved spatial phase *η* for our chosen set of parameters corresponding to the spatial position of the Turing pattern. In the language of dynamical systems, *η* parameterizes a continuous set of attractors for this dissipative system.

**Fig. 6:**
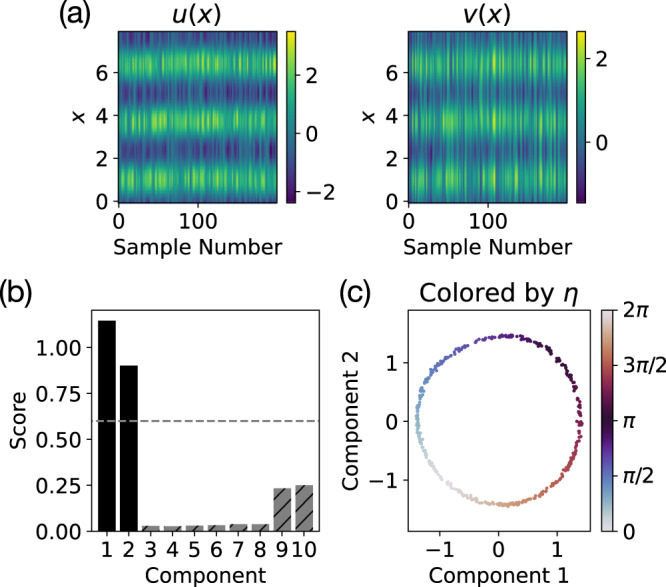
Identifying the conserved spatial phase for the oscillating Turing pattern system. **a** An example trajectory, with randomly sampled states *u*(*x*) and *v*(*x*) plotted, illustrates the high dimensional nature of the problem. **b** The heuristic score (with cutoff 0.6) identifies two relevant components, but on further examination, **c** we see that there is just a single conserved angle, corresponding to the spatial phase *η* of the Turing pattern, that needs to be embedded in two dimensions due to its topology.

Our method successfully identifies the spatial phase *η* but embeds the angle as a circle in a 2D embedding space (Fig. 6)—a result of the periodic topology of *η*. While this shows that the number of relevant components determined by our heuristic score may not always match the true manifold dimensionality, such cases are often easily identified by examining the components directly (Fig. 6c) or by cross checking with an intrinsic dimensionality estimator^27^. A linear fit of the two relevant components with $\cos \eta$ ($\sin \eta$) has *R*^2^ = 0.9991 (*R*^2^ = 0.9997) and *ρ* = 0.9993 (*ρ* = 0.9992). This example both tests our method on a high dimensional phase space and demonstrates how our approach can be applied to dissipative systems to study long term behavior.

#### Korteweg–De Vries equation

**Fig. 7:**
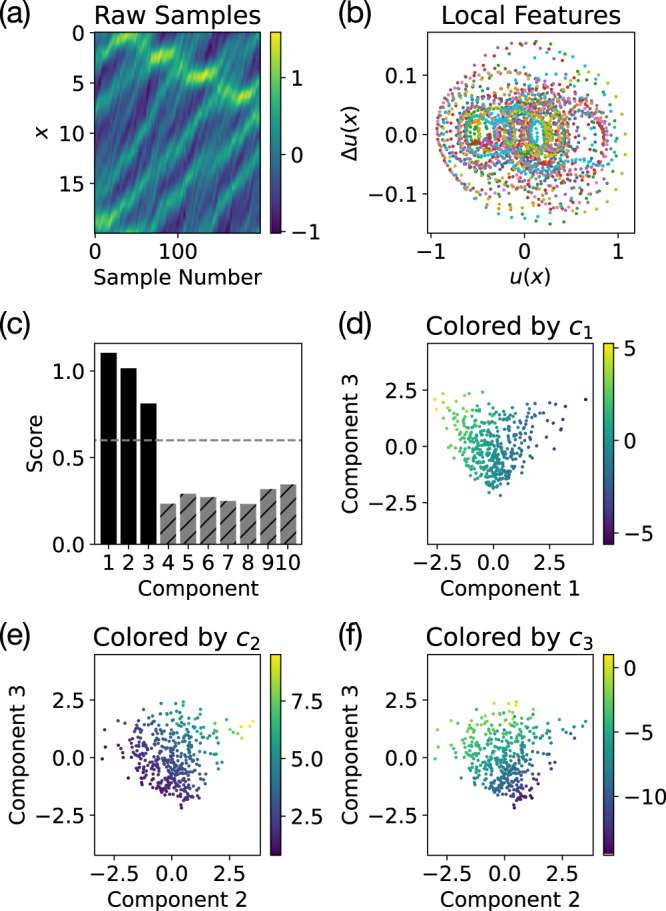
Identifying three local conserved quantities of the Korteweg–De Vries (KdV) equation. **a** An example trajectory from the KdV dataset shows the high dimensional raw sampled states *u*(*x*). **b** To focus on local conserved quantities, we extract a distribution of the local features *u*(*x*), Δ*u*(*x*) from the raw states, removing the explicit spatial label. The plot shows the local feature distributions for a few sample states. **c** The heuristic score (with cutoff 0.6) correctly identifies three relevant components corresponding to **d**–**f** the three local conserved quantities (Eq. (14)).

For many spatiotemporal dynamical systems, the conservation laws are local in nature. Locality can significantly simplify the analysis of the conserved quantities and suggests a way to restrict the type of conserved quantities identified by our method. Specifically, we can adapt our approach to focus on local conserved quantities by replacing the raw states (Fig. 7a) by a distribution of local features (Fig. 7b), removing the explicit spatial label and providing a fully translation invariant representation of the state. Then, instead of using the Euclidean metric in the original phase space, we use the energy distance^28,29^ between the distributions of local features as the ground metric for optimal transport.

To demonstrate this method for identifying local conserved quantities, we consider the Korteweg–De Vries (KdV) equation

13 $$\frac{\partial u}{\partial t}=-\frac{{\partial }^{3}u}{\partial {x}^{3}}-6u\frac{\partial u}{\partial x}.$$

The KdV equation is fully integrable^4^ and has infinitely many conserved quantities^3^, the most robust of which are the most local conserved quantities expressible in terms of low order spatial derivatives. To focus on these robust local conserved quantities, we use finite differences (i.e. *u*(*x*), Δ*u*(*x*) = *u*(*x* + Δ*x*) − *u*(*x*), Δ^2^*u*(*x*), …) as our local features, allowing us to restrict the spatial derivative order of the identified conserved quantities. In this experiment, we only take *u*(*x*) and Δ*u*(*x*), meaning that the identified local conserved quantities will only contain up to first order spatial derivatives. For the KdV equation, there are three such local conserved quantities:

14 $${c}_{1}=	\int \nolimits_{0}^{l}u\,{{{{{{{\rm{d}}}}}}}}x\\ {c}_{2}=	\int\nolimits_{0}^{l}{u}^{2}\,{{{{{{{\rm{d}}}}}}}}x\\ {c}_{3}=	\int \nolimits_{0}^{l}\left[{u}^{3}-\frac{1}{2}{\left(\frac{\partial u}{\partial x}\right)}^{2}\right]\,{{{{{{{\rm{d}}}}}}}}x,$$

where *c*_1_ and *c*_2_ are often identified as “momentum” and “energy”, respectively^3^. These local conserved quantities also have direct analogues in generalized KdV-type equations, hinting at their robustness^30^.

Our method successfully identifies three relevant components (Fig. 7c) corresponding to (d–f) the three local conserved quantities (Eq. (14)). Linear fits of these components to *c*_1_, *c*_2_, and *c*_3_ have rank correlations *ρ* = 0.995, 0.994, and 0.985, respectively. This result shows how our approach can be adapted to incorporate known structure, such as locality and translation symmetry, in applications to complex high dimensional dynamical systems.

## Discussion

We have proposed a non-parametric manifold learning method for discovering conservation laws, tested our method on a wide variety of dynamical systems—including complex chaotic systems with multiple phases and high dimensional spatiotemporal dynamics—and also shown how to adapt our approach to incorporate additional structure such as locality and translation symmetry. While our experiments use dynamical systems with known conserved quantities in order to validate our approach, our method does not require any a priori information about the conserved quantities. Our method also does not assume or construct an explicit model for the system nor require accurate time information like previous approaches^12,13^, only relying on the ergodicity of the dynamics modulo the conservation laws (Section “Ergodicity and physical measures”). As a result, our approach is also quite robust to measurement noise and can often deal with missing information such as a partially observed phase space (Fig. 2e–h, Fig. 3e–h, Supplementary Note 5).

Compared with recently proposed deep learning-based methods^15,16^, our approach is substantially more interpretable since it relies on explicit geometric constructions and well-studied manifold learning methods that directly determine the geometry of the shape space ${{{{{{{\mathcal{C}}}}}}}}$ and, therefore, the identified conserved quantities. This is reflected in our ability to explicitly derive the expected result for the simple harmonic oscillator (Section “Analytic result for the simple harmonic oscillator”), as well as in the identified conserved quantities in many of our experiments. For example, the embedding of the semi-major axis vector in the planar gravitational dynamics experiment (Section “Planar gravitational dynamics”) stems directly from the elliptical geometry of the orbits and their orientation in phase space, which is captured by the Euclidean ground metric and lifted into shape space by optimal transport. Our method also correctly captures the subtleties of the double pendulum system (Section “Double pendulum”) by providing an embedding that shows both a 1D manifold at high energies and a 2D manifold at low energies—a difficult prospect for deep learning approaches that try to explicitly fit conserved quantities. In addition, we empirically find that our method outperforms existing direct fitting approaches^15,16^. See Supplementary Note 6 for a comparison benchmark using our planar gravitational dynamics dataset.

Our manifold learning approach to identifying conserved quantities provides a new way to analyze data from complex dynamical systems and uncover useful conservation laws that will ultimately improve our understanding of these systems as well as aid in developing predictive models that accurately capture long term behavior. While our method does not provide explicit symbolic expressions for the conserved quantities (which may not exist in many cases), we do obtain a full set of independent conserved quantities, meaning that any other conserved quantity will be a function of the discovered ones. Our method also serves as a strong non-parametric baseline for future methods that aim to discover conservation laws from data. Finally, we believe that similar combinations of optimal transport and manifold learning have the potential to be applied to a wide variety of other problems that also rely on geometrically characterizing families of distributions, and we hope to investigate such applications in the near future.

## Methods

Our proposed approach uses manifold learning to identify and embed the manifold of phase space isosurfaces sampled by the trajectories of a dynamical system. In particular, we compute a diffusion map over a set of trajectories, each of which samples a particular phase space isosurface (Fig. 1a). The pairwise distances between these trajectories are given by the 2-Wasserstein distance (Fig. 1b), providing the metric structure necessary for applying diffusion maps (Fig. 1c). The manifold embedding extracted by the diffusion map corresponds directly to the space of conserved quantities (Fig. 1d). Note that this type of analysis does not require knowledge of the equations of motion (Eq. (15)) and makes no direct reference to time.

### Dynamical systems

Consider a dynamical system with states ${{{{{{{\bf{x}}}}}}}}\in {{{{{{{\mathcal{M}}}}}}}}$ that live in a *d*-dimensional phase space ${{{{{{{\mathcal{M}}}}}}}}$ and evolve in time according to a system of first order ODEs

15 $$\frac{{{{{{{{\rm{d}}}}}}}}{{{{{{{\bf{x}}}}}}}}}{{{{{{{{\rm{d}}}}}}}}t}={{{{{{{\bf{F}}}}}}}}({{{{{{{\bf{x}}}}}}}})$$

with *n* conserved quantities *G*_1_(**x**), …, *G*_*n*_(**x**).

#### Conserved quantities and phase space isosurfaces

A conserved quantity *G*_*i*_(**x**) is a function of the system state **x** that does not change over time, i.e.

16 $$\frac{{{{{{{{\rm{d}}}}}}}}{G}_{i}({{{{{{{\bf{x}}}}}}}}(t))}{{{{{{{{\rm{d}}}}}}}}t}=0,$$

but may vary across different initial conditions. As a result, along a particular trajectory **x**(*t*), the *n* conserved quantities form a set of constraints

17 $${G}_{i}({{{{{{{\bf{x}}}}}}}})={c}_{i},\quad i \in \{1,2,\ldots,n\}$$

which depend on the values of the conserved quantities **c** = {*c*_1_, *c*_2_, …, *c*_*n*_}. This set of constraint equations restricts the trajectory to lie in a phase space isosurface ${{{{{{{{\mathcal{X}}}}}}}}}_{{{{{{{{\bf{c}}}}}}}}}\subseteq {{{{{{{\mathcal{M}}}}}}}}$ with dimension *d* − *n*. In fact, if any point of a trajectory lies on the isosurface ${{{{{{{{\mathcal{X}}}}}}}}}_{{{{{{{{\bf{c}}}}}}}}}$, then all other points from the trajectory will lie on the same isosurface.

By studying the variations in shape of these isosurfaces, we are able to directly characterize the space of conserved quantities. In particular, consider the manifold ${{{{{{{\mathcal{C}}}}}}}}$ formed by the isosurfaces ${{{{{{{{\mathcal{X}}}}}}}}}_{{{{{{{{\bf{c}}}}}}}}}$ in shape space. This manifold ${{{{{{{\mathcal{C}}}}}}}}$ is parameterized by the conserved quantities **c**. Therefore, by analyzing ${{{{{{{\mathcal{C}}}}}}}}$ using manifold learning, we can extract the conservation laws of the underlying dynamical system.

Note that we are using the term “manifold” here rather loosely. While ${{{{{{{\mathcal{C}}}}}}}}$ may be a true manifold in many cases, it is also possible for ${{{{{{{\mathcal{C}}}}}}}}$ to have non-manifold structure (e.g. see our double pendulum experiment in the section “Double pendulum”).

#### Ergodicity and physical measures

To uniquely identify the isosurface associated with each trajectory, we must make several additional assumptions that will allow us to treat the set of points making up each trajectory as samples from an ergodic invariant measure on the corresponding isosurface. Specifically, we assume that, for each trajectory **x**(*t*) with conserved quantities **c**, the dynamical system (Eq. (15)) admits a physical measure^31^ that is ergodic on the isosurface ${{{{{{{{\mathcal{X}}}}}}}}}_{{{{{{{{\bf{c}}}}}}}}}$ and is defined by

18 $${\mu }_{{{{{{{{\bf{c}}}}}}}}}=\mathop{\lim }\limits_{T\to \infty }\frac{1}{T}\int\nolimits_{0}^{T}{\delta }_{{{{{{{{\bf{x}}}}}}}}(t)}\,{{{{{{{\rm{d}}}}}}}}t,$$

where *δ*_**x**(*t*)_ is the Dirac measure centered on **x**(*t*). This ensures that trajectories with the same conserved quantities will sample the same distribution on the same isosurface, allowing us to use the distribution sampled by each trajectory as a proxy for the corresponding isosurface. For more details about this assumption, see Supplementary Note 7.4.

In practice, the sampled distribution may be lower dimensional than the corresponding isosurface if some of the conserved quantities do not vary in the dataset and instead correspond to fixed constraints, or if the dynamical system is dissipative. In the former case, this does not affect our ability to uniquely identify a distribution with an isosurface and its corresponding set of conserved quantities, meaning that we are able to apply this approach even if the provided phase space is much larger than the intrinsic phase space of the dynamical system. In the latter case, the dissipative nature of the system may cause information about conservation laws relevant during the transient portion of the dynamics to be lost, but we are still able to use our approach to identify conserved quantities relevant for the long term behavior of the system.

### Wasserstein metric

To analyze the isosurface shape space manifold ${{{{{{{\mathcal{C}}}}}}}}$—i.e. the manifold of conserved quantities—using manifold learning methods, we need to place some structure on the points ${{{{{{{{\mathcal{X}}}}}}}}}_{{{{{{{{\bf{c}}}}}}}}}\in {{{{{{{\mathcal{C}}}}}}}}$. Having associated each isosurface ${{{{{{{{\mathcal{X}}}}}}}}}_{{{{{{{{\bf{c}}}}}}}}}$ with a corresponding distribution defined by an ergodic physical measure *μ*_**c**_, we choose to lift the Euclidean metric on the phase space into the space of distributions using the 2-Wasserstein metric from optimal transport

19 $${W}_2(\mu_{{{{{{\mathbf{c}}}}}}},\mu_{{{{{{\mathbf{c}}}}}}^{\prime}})=\left(\mathop{\inf} \limits_{\pi \in {{\Pi}}(\mu_{{{{{{\mathbf{c}}}}}}},\mu_{{{{{{\mathbf{c}}}}}}^{\prime}})} {\int}\, {c}({{{{{\mathbf{x}}}}}},{{{{{\mathbf{y}}}}}})\,{{{{{\mathrm{d}}}}}}\pi({{{{{\mathbf{x}}}}}},{{{{{\mathbf{y}}}}}})\right)^{1/2},$$

where the cost function *c*(**x**, **y**) = ∥**x** − **y**∥^2^ is the squared Euclidean distance, and $\pi \in {{\Pi }}({\mu }_{{{{{{{{\bf{c}}}}}}}}},{\mu }_{{{{{{{{{\bf{c}}}}}}}}}^{{\prime} }})$ is a valid transport map between *μ*_**c**_ and ${\mu }_{{{{{{{{{\bf{c}}}}}}}}}^{{\prime} }}$^17^.

For discrete samples, the 2-Wasserstein distance between two sets of sample points {**x**_1_, **x**_2_, …, **x**_*S*_} and {**y**_1_, **y**_2_, …, **y**_*S*_} is defined as

20 $${W}_{2}={\left(\mathop{\min }\limits_{T}\mathop{\sum}\limits_{i,j}{T}_{ij}{C}_{ij}\right)}^{1/2},$$

where the cost matrix *C*_*ij*_ = ∥**x**_*i*_ − **y**_*j*_∥^2^, and the transport matrix *T* is subject to the constraints

21 $$\begin{array}{ll}&{T}_{ij}\ge 0,\,\forall i,j\\ &\mathop{\sum}\limits_{j}{T}_{ij}=1\\ &\mathop{\sum}\limits_{i}{T}_{ij}=1.\end{array}$$

To efficiently compute an entropy regularized form of this optimization problem, we use the Sinkhorn algorithm^32^ and estimate the Wasserstein distance as a debiased Sinkhorn divergence^33^.

One important subtlety in this construction is the choice of the ground metric for optimal transport. We use a Euclidean metric on the phase space, which implicitly imposes a choice of units to make the phase space dimensionless. In fact, there is no canonical choice for the ground metric, and different choices result in different Wasserstein metrics on the shape space. While, in theory, information about all conserved quantities will be embedded in the resulting distance matrix regardless of the choice of metric, the metric ultimately determines how easy it is to access this information. For example, when multiple conserved quantities are present, the relative effect of each conserved quantity on the computed Wasserstein distances will determine how prominent each conserved quantity is and how easily it is identified using manifold learning. To partially mitigate this issue and improve consistency, we normalize each component of our data to have a maximum absolute value of 1 before computing the pairwise Wasserstein distances.

Finally, using the Wasserstein distance provides our approach with a tremendous amount of robustness (Supplementary Note 5), but also makes it susceptible to certain kinds of sampling inhomogeneity. See Supplementary Note 7.3 for a more detailed discussion of this trade off.

### Diffusion maps

Using the structure provided by the Wasserstein metric, we then use diffusion maps to generate an embedding for ${{{{{{{\mathcal{C}}}}}}}}$. The diffusion map manifold learning method uses a spectral embedding algorithm applied to an affinity matrix to construct a low dimensional embedding of the data manifold^18,19^. Using the pairwise Wasserstein distances *W*_2_(*μ*_*i*_, *μ*_*j*_) computed from discrete samples provided by the trajectory data (Eq. (20)), we first construct a kernel matrix using a Gaussian kernel

22 $${K}_{ij}=\exp (-{W}_{2}{({\mu }_{i},{\mu }_{j})}^{2}/\epsilon )$$

and then scale it to form an affinity matrix for our spectral embedding

23 $${M}_{ij}={K}_{ij}/{({D}_{i}{D}_{j})}^{\alpha },$$

where *D*_*i*_ = ∑_*k*_*K*_*ik*_, and *α* is a hyperparameter. The spectral embedding algorithm then takes this affinity matrix and constructs a normalized graph Laplacian

24 $${L}_{ij}={I}_{ij}-{M}_{ij}/\mathop{\sum}\limits_{k}{M}_{ik},$$

where *I* is the identity matrix. The eigenvectors **v**_*i*_ corresponding to the smallest eigenvalues *λ*_*i*_ ≥ 0 (excluding *λ*_0_ = 0) of the Laplacian then provide an approximate low dimensional embedding of the manifold of conserved quantities ${{{{{{{\mathcal{C}}}}}}}}$. In our experiments, we set *α* = 1 so that the Laplacian computed by the spectral embedding algorithm approximates the Laplace–Beltrami operator^18^.

To estimate the dimensionality of ${{{{{{{\mathcal{C}}}}}}}}$ and to choose which eigenvectors **v**_*i*_ to include in our embedding, we use a heuristic score that combines a measure of relevance, given by a length scale computed from the Laplacian eigenvalues, with a previously suggested measure of “unpredictability” for minimizing redundancy^34^ (alternative approaches also exist^35,36^). To construct our embedding, we only include the Laplacian eigenvectors with score above a chosen cutoff value and discard the rest as either noise or redundant embedding components. To determine the cutoff, we perform a sweep of the cutoff value looking for robust ranges and find that a cutoff of 0.6 works well across all of our experiments, which consist of a wide variety of datasets and dynamical systems. See Supplementary Note 1 for more details.

### Supplementary information

Supplementary Information

## Data Availability

The data in this study can be generated using the publicly available data generation scripts provided at https://github.com/peterparity/conservation-laws-manifold-learning. An archived version has also been deposited in the Zenodo database 10.5281/zenodo.8144481^37^.

## Code availability

All the code necessary for reproducing our results is publicly available at https://github.com/peterparity/conservation-laws-manifold-learning. An archived version has also been deposited in the Zenodo database 10.5281/zenodo.8144481^37^.
