# Supplementary material for: Discovering conservation laws using optimal transport and manifold learning
Source: Nat Commun. 2023 Aug 7;14:4744. doi: 10.1038/s41467-023-40325-7 (PMC10406953; doi:10.1038/s41467-023-40325-7)
Supplement: Supplementary file 1 — Supplementary Information [file 41467_2023_40325_MOESM1_ESM.pdf]

Supplementary Information: Discovering Conservation Laws using Optimal Transport and Manifold Learning

Peter Y. Lu, Rumen Dangovski, Marin Soljačić

Supplementary Note 1 Heuristic Score for a Minimal Diffusion Maps Embedding

Traditionally, diffusion maps [19] and Laplacian eigenmaps [18] leave the embedding dimension n as a hyperparameter and simply use the eigenvectors corresponding to the n smallest eigenvalues to construct the embedding. In practice, the embedding dimension n is often chosen for convenience (e.g. in visualization applications) or by examining the eigenvalues λ_i and looking for a sharp increase in the magnitude of the eigenvalues that would separate the signal from the noise. Because identifying the number of conservation laws is an important step in our approach, we refine this heuristic by directly computing an approximate length scale

$$l_i = \sqrt{-\epsilon / \log(1 - \lambda_i)}, \tag{S1}$$

where ϵ is the scale factor from the Gaussian kernel (Eq. 22) used to construct the Laplacian matrix L. We derive this length scale by considering the normalized kernel $I - L$ to be an approximation of the heat kernel $\exp(\epsilon\Delta)$, implying that the length scales l_i associated with the Laplace–Beltrami operator Δ are given by

$$\exp(\epsilon\Delta) = I - L \implies \exp(-\epsilon/l_i^2) = 1 - \lambda_i. \tag{S2}$$

We then divide by l_1 to obtain the relative length scale

$$\frac{l_i}{l_1} = \sqrt{\frac{\log(1-\lambda_1)}{\log(1-\lambda_i)}}, \tag{S3}$$

which can be used as a heuristic measure of relevance—components with a small relative length scale are more likely to be noise. Compared with directly using the eigenvalues λ_i, we find this heuristic to be less sensitive to the choice of ϵ in the kernel.

In addition to noise, there is the common problem of redundant embedding components that stem from the structure of the Laplacian operator: higher order modes of previous eigenvectors often appear before more informative eigenvectors corresponding to new manifold directions. This problem is clearly illustrated in the planar gravitational dynamics experiment (Sec. 2.2.3), where components 3, 4, and 5 are all redundant with components 1 and 2 but component 6 is a new and relevant conserved quantity (Figs. 1d–f). To address this issue, the key observation is that, while all components of the diffusion map are linearly independent, redundant components are still predictable (via a nonlinear function) from previous components. Therefore, we require a measure of "unpredictability" that allows us to identify redundancies. We choose the heuristic m_i proposed by Pfau and Burgess [34] that uses a nearest neighbor estimator (using 5 nearest neighbors) to determine whether a new embedding component is too predictable and therefore redundant. Alternative methods for dealing with these redundant components, also called repeated eigendirections or higher harmonics, have been proposed that use local linear regression to detect redundant components [35] or adapt the diffusion kernel to be anisotropic for chosen components [36].

Our final heuristic score (Fig. 1c)

$$s_i = m_i l_i / l_1 \tag{S4}$$

is the product of the relative length scale l_i/l_1 (Fig. 1a) and the unpredictability measure m_i (Fig. 1b). We find this simple combined score performs well for identifying relevant embedding components by removing both noise components as well as redundant components.

1.1 Choosing a Score Cutoff

To use the heuristic score to identify the number of conserved quantities and construct a minimal embedding, we require a score cutoff to separate relevant components that we keep in our embedding from irrelevant components that we discard. To choose this cutoff, we sweep cutoff values in the interval $[0, 1]$, compute the embedding size (i.e. the number of relevant components) based on the chosen cutoff, and then examine the result to identify a robust value for the cutoff (Fig. 2). Specifically, we look for wide plateaus in the embedding size that indicate robustness to the value of the cutoff and find that a cutoff of 0.6 works well in all of our experiments. In practice, we would treat a cutoff of 0.6 as a good starting point but recommend analyzing a range of cutoff values to find a robust choice of cutoff, as illustrated in Fig. 2.

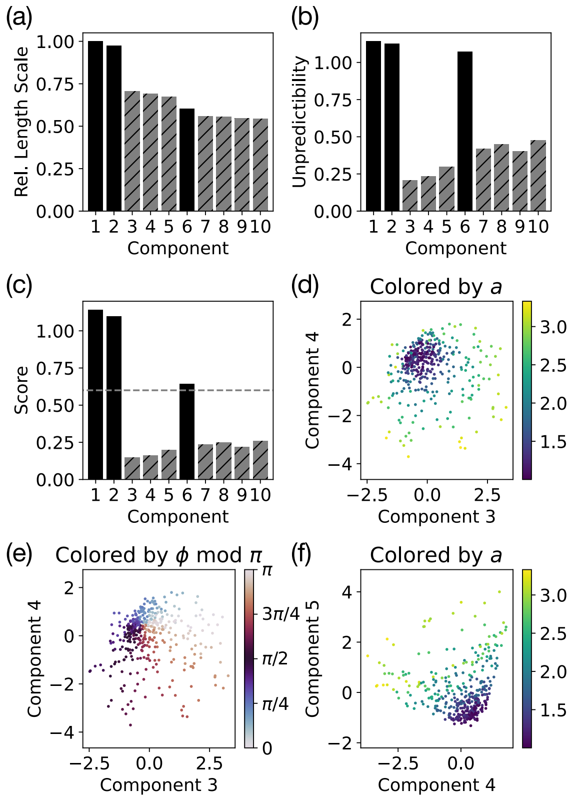

Supplementary Figure 1 Breakdown of the heuristic score and illustration of redundant embedding components from the planar gravitational dynamics experiment. (a) The relative length scale l_i/l_1 for each embedding component is computed from the corresponding eigenvalue λ_i of the Laplacian matrix (Eq. S1). (b) The unpredictability measure m_i for each component is computed using a nearest neighbor estimator [34]. (c) The combined score $m_i l_i/l_1$ is the product of the relative length scale and the unpredictability measure. (d–f) The components 3, 4, and 5 are identified by the unpredictability measure as redundant. If we examine these three components, we find that they together embed a second order angular mode of components 1 and 2 (Figs. 4d,e). In particular, the embedding is shaped like the surface of a cone with the height (or radial distance) roughly corresponding to the semi-major axis a and the angle around the cone corresponding to ϕ mod π, a second order mode of the orientation angle ϕ.

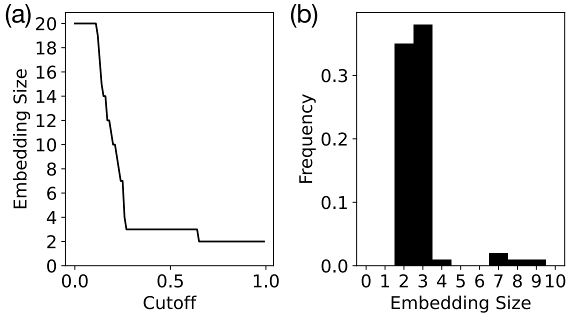

Supplementary Figure 2 Example from the planar gravitational dynamics experiment of identifying the number of conserved quantities (i.e. the embedding size). (a) Sweeping the cutoff value from 0 to 1, we find plateaus indicating robustness at embedding size 2 and 3. Note that there is a spurious plateau at the maximum embedding size 20. (b) A histogram of the embedding sizes confirms that the number of conserved quantities is likely to be 3.

Supplementary Note 2 Additional Method Details

All of the code necessary for generating our datasets, applying our method, and reproducing our results is available at https://github.com/peterparity/conservation-laws-manifold-learning.

2.1 Sinkhorn Algorithm

2.1.1 Entropy Regularized Optimal Transport

We compute an approximate 2-Wasserstein distance using the Sinkhorn algorithm [32], which solves an entropy regularized relaxation of the optimal transport problem

$$\widetilde{W}_2(\{\mathbf{x}_i\}, \{\mathbf{y}_j\}) = \left(\min_T \sum_{i,j} T_{ij} C_{ij} - \gamma\, h(T) \right)^{1/2}, \tag{S5}$$

where the cost matrix $C_{ij} = \|\mathbf{x}_i - \mathbf{y}_j\|^2$ and the entropy $h(T) = -\sum_{i,j} T_{ij} \log T_{ij}$. $\widetilde{W}_2$ reduces to the exact 2-Wasserstein distance W_2 (Eq. 20) as $\gamma \to 0$. For $\gamma > 0$, the entropy regularization introduces a smoothing bias that manifests as a nonzero "self-distance" $\widetilde{W}_2(\{\mathbf{x}_i\}, \{\mathbf{x}_j\}) > 0$. This can be corrected by instead using the Sinkhorn divergence [29, 33]

$$\overline{W}_2 = \left(\widetilde{W}_2(\{\mathbf{x}_i\}, \{\mathbf{y}_j\})^2 - \frac{\widetilde{W}_2(\{\mathbf{x}_i\}, \{\mathbf{x}_j\})^2 + \widetilde{W}_2(\{\mathbf{y}_i\}, \{\mathbf{y}_j\})^2}{2} \right)^{1/2} \tag{S6}$$

as our estimate for the 2-Wasserstein distance, which explicitly subtracts off this self-distance. In our experiments, we use a convergence threshold of $\varepsilon = 0.01$ and a decaying entropy regularization parameter γ that starts at 10.0 and decays by a factor of 0.995 at each step until it reaches a target of 0.1.

Note that the Sinkhorn algorithm is a general approach for solving entropy-regularized optimal transport and is equally good at approximating a 1-Wasserstein distance (or Earth mover's distance). We experimented with using 1-Wasserstein vs. 2-Wasserstein distances and did not find a significant difference in the performance of our method.

2.1.2 Time Complexity

With S samples per trajectory, the Sinkhorn algorithm solves the entropy regularized optimal transport problem in $\mathcal{O}(S^2 \log S/\varepsilon^2)$ time for an ε-accurate solution [S1, S2] using $\mathcal{O}(S)$ space (without explicit storing the cost matrix C [S3]). Therefore, the time complexity of computing approximate Wasserstein distances for all pairs of trajectories is $\mathcal{O}(N^2 S^2 \log S/\varepsilon^2)$ for a dataset containing N total trajectories. This computation is currently the performance bottleneck of our approach (Supplementary Note 7.1) but is easily parallelized over multiple GPUs using the OTT-JAX library [S3].

2.2 Diffusion Maps

2.2.1 Choice of Manifold Learning Method

Unlike many standard manifold learning applications, our input to the manifold learning method is not a set of points in Euclidean space but rather a pairwise Wasserstein distance matrix. Many popular methods, such as local linear embedding [S4] and local tangent space alignment [S5], explicitly require the data to be embedded in a Euclidean space and so are not applicable for our problem. In addition to diffusion maps, we also experimented with Isomap [S6], which can also take a distance matrix as input. However, we found Isomap embeddings to be noisier and less reliable than diffusion map embeddings (e.g. see Fig. 3). Diffusion maps [19] or Laplacian eigenmaps [18] also provide an effective way to estimate manifold dimensionality and choose relevant embedding components (Supplementary Note 1).

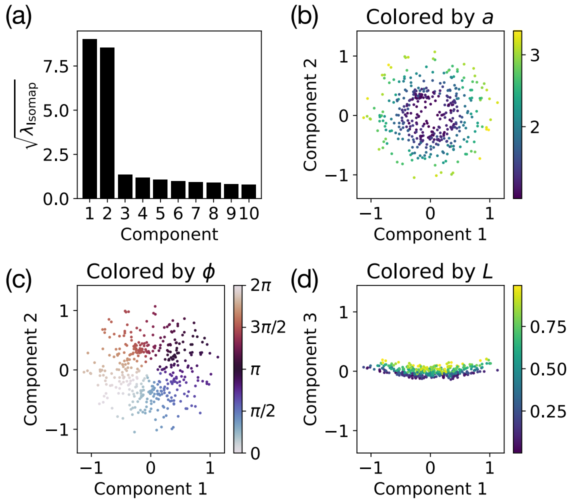

Supplementary Figure 3 Identifying conserved quantities for planar gravitational dynamics using Isomap. (a) When applying Isomap, the effective length scale $\sqrt{\lambda_{\text{Isomap}}}$ can be used to estimate manifold dimensionality but it only clearly identifies two out of the three conserved quantities. (b, c) Similarly to the diffusion map embedding, Isomap components 1 and 2 embed the semi-major axis vector $\mathbf{a}$ with magnitude $a = -1/2E$ related to the energy and orientation given by the angle ϕ. These components have high rank correlation $\rho = 0.994$ ($\rho = 0.992$) with $a \cos\phi$ ($a \sin\phi$). (d) Component 3 roughly corresponds to the angular momentum L but is noisier than the embedding identified by diffusion maps (Fig. 4f) and has a much lower rank correlation $\rho = 0.723$ with L.

2.2.2 Gaussian Kernel Width

The primary hyperparameter in our diffusion maps algorithm is the width parameter ϵ of the Gaussian kernel (Eq. 22). Generally speaking, we should choose ϵ to be large enough to avoid noise induced by sparse sampling but small enough for the true heat kernel to be well approximated by the Gaussian kernel [18]. We choose ϵ such that the corresponding standard deviation $\sigma = \sqrt{\epsilon/2}$ of the Gaussian kernel is equal to the maximum distance to the kth nearest neighbor.

In practice, especially when the relevant embedding components are well separated from the noise in terms of length scale (Eq. S1), we find the diffusion map to be fairly insensitive to the choice of k, which we generally set to $k = 20$ nearest neighbors. The only exception is for the planar gravitational dynamics dataset, where we use $k = 200$. This is because the angular momentum—the least prominent of the three conserved quantities—has a slightly poorer reconstruction for $k = 20$ ($\rho = 0.910$) and gets pushed back to component 10 of the embedding. While it is still clearly identifiable from noise, it requires a lower heuristic score cutoff of around ~ 0.4 and is easier to miss.

2.2.3 Noise Robustness

To improve the noise robustness of our diffusion map, we follow Karoui and Wu [S7] and replace the diagonal of the affinity matrix M (Eq. 23) with zeros, i.e.

$$M^*_{ij} = M_{ij} - M_{ii} I_{ij}, \tag{S7}$$

before constructing the Laplacian matrix L. Because this induces an overall shift in the eigenvalues of the Laplacian that interacts poorly with our length scale heuristic (Eq. S1), we correct for this by subtracting off the normalized mean shift

$$s = \frac{1}{N} \sum_{i=1}^{N} \left(M_{ii} / \sum_j M_{ij} \right) \tag{S8}$$

from the Laplacian matrix L to obtain the corrected Laplacian

$$L^*_{ij} = L_{ij} - sI_{ij}, \tag{S9}$$

which we use to generate our embeddings.

2.2.4 Time Complexity

For a fixed number of embedding dimensions and a dense kernel matrix K with $N \times N$ entries (N being the number of trajectories), diffusion maps have time complexity $\mathcal{O}(N^2)$ and space complexity $\mathcal{O}(N^2)$. In our experiments, computing the diffusion map is very fast with run times under one second for every dataset we tested.

2.2.5 Out-of-Sample Embedding

One complication of manifold learning methods like diffusion maps is that they do not provide an explicit way to embed new out-of-sample data. A naive approach would be to rerun the diffusion map algorithm on a combined dataset consisting of the original data used to create the embedding and the new data. However, this does not retain the original embedding and, in some cases, may be computationally prohibitive. A popular approach that does retain the original embedding is the Nyström method [S8], which embeds a new point using its pairwise distances with the original data and scales as $\mathcal{O}(N)$. For even faster embedding, landmark diffusion maps offer a significant speed-up by choosing a small subset of $M \ll N$ landmark points to use during Nyström out-of-sample embedding [S9]. This also has the added benefit of a reduced memory footprint, since only the M landmark points need to be retained for embedding.

Supplementary Note 3 Langevin Harmonic Oscillator

To demonstrate our method on a simple example of a dynamical system with approximately conserved quantities at short time scales but no true conserved quantities, we consider an under-damped harmonic oscillator that is weakly coupled to a heat bath. The resulting dynamics are governed by the Langevin equations

$$\begin{aligned} \frac{\mathrm{d}q}{\mathrm{d}t} &= p \\ \frac{\mathrm{d}p}{\mathrm{d}t} &= -q - \gamma p + \xi(t), \end{aligned} \tag{S10}$$

where the damping $\gamma = 10^{-2}$. $\xi(t)$ is a Gaussian random process (i.e. Brownian motion) with zero mean and $\langle \xi(t)\xi(t') \rangle = 2\gamma\tau\,\delta(t-t')$. We set the temperature of the heat bath to be $\tau = 2 \times 10^{-2}$. At short times $t \ll \gamma^{-1}$ and $t \ll (\gamma\tau)^{-1/2}$, the energy $E = (q^2 + p^2)/2$ is approximately conserved. At long times $t \gg \gamma^{-1}$, all trajectories will sample a stationary distribution with variance $\langle q^2 \rangle_0 = \tau$ and no conserved quantities.

We generate four datasets for this system, each with 200 trajectories and 200 samples over different periods of time, i.e. $t \in [0, T]$ with sampling time $T \in \{2 \times 10^1, 2 \times 10^2, 2 \times 10^3, 2 \times 10^4\}$. This allows us to study how changing the sampling time T—the time scale over which we identify approximately conserved quantities—changes the embedding produced by our approach. Over short time scales, the time-averaged energy $\langle E \rangle$ is still a distinguishing feature of the trajectories and acts as an approximately conserved quantity (Figs. 4a–d). Over longer time scales, the energy becomes less and less relevant until, finally, the system reaches a stationary distribution (Figs. 4e–h).

3.1 Separation of Time Scales and Dependence on γ and τ

The energy of the Langevin harmonic oscillator is only conserved over time scales T much less than the time scales associated with dissipation γ^{-1} and random forcing $(\gamma\tau)^{-1/2}$. However, this approximate conservation law is only meaningful if the energy is roughly conserved over a time scale T much greater than the period of oscillation, which is of order one in our units. In other words, approximate conservation laws only appear when there is a separation of time scales between fast conservative dynamics (e.g. the oscillations of our harmonic oscillator) and slow non-conservative dynamics (e.g. dissipation and forcing).

Thus, for $\gamma \ll 1$ and $\tau \ll 1$, we expect to have an approximately conserved energy at intermediate time scales $1 \ll T \ll \gamma^{-1}$ and $T \ll (\gamma\tau)^{-1/2}$. As $\gamma, \tau \to 1$, there is no longer an intermediate time scale for which the approximate conservation law holds. This is precisely what we see when we apply our method for identifying conservation laws to Langevin harmonic oscillators with varying $\gamma \in \{10^{-3}, 10^{-2}, 10^{-1}\}$ and $\tau \in \{2 \times 10^{-3}, 2 \times 10^{-2}, 2 \times 10^{-1}\}$ using sampling times $T \in \{2 \times 10^1, 2 \times 10^2, 2 \times 10^3, 2 \times 10^4\}$ (Fig. 5).

The rank correlation ρ (Fig. 5a) shows the alignment of the first embedding component with the time-averaged energy $\langle E \rangle$, while the prominence $s_1 - \max(\{s_i\}_{i>1})$ (Fig. 5b) of the heuristic score shows how well distinguished the first component is from the remaining noisy components. We find that for small $\gamma \leq 0.01$ and $\tau \leq 0.02$, our approach successfully identifies the approximately conserved energy at intermediate time scales $T = 20$ or $T = 200$ (as shown by the high rank correlation and high score prominence). For longer time scales or larger γ and τ, the approximate conservation law no longer holds and so our method identifies no conserved quantities (as shown by the low score prominence).

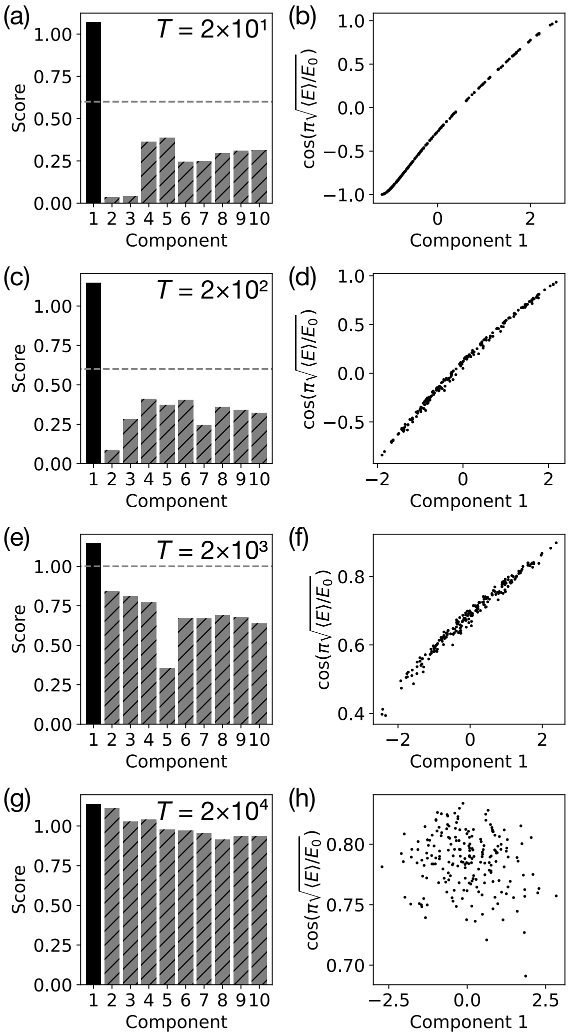

Supplementary Figure 4 Identifying conserved quantities for the Langevin harmonic oscillator over varying sampling times T. (a) Over a short time scale $T = 2 \times 10^1$, the time-averaged energy $\langle E \rangle$ still clearly distinguishes the different trajectories, so our approach identifies a single (approximately) conserved quantity that corresponds to $\langle E \rangle$. (b) Component 1 of the embedding is highly correlated with $\langle E \rangle$ and still matches well with the theoretical result for the simple harmonic oscillator (Eq. 5). (c,d) Over a slightly longer time scale $T = 2 \times 10^2$, we see a very similar result with a noisier fit between component 1 and $\langle E \rangle$. (e,f) For $T = 2 \times 10^3$, we start to see more ambiguity, with the identified component becoming significantly less prominent and an even noisier fit. (g,h) Over a long time scale $T = 2 \times 10^4$, the system has settled into a stationary distribution and no longer has any even approximately conserved quantities.

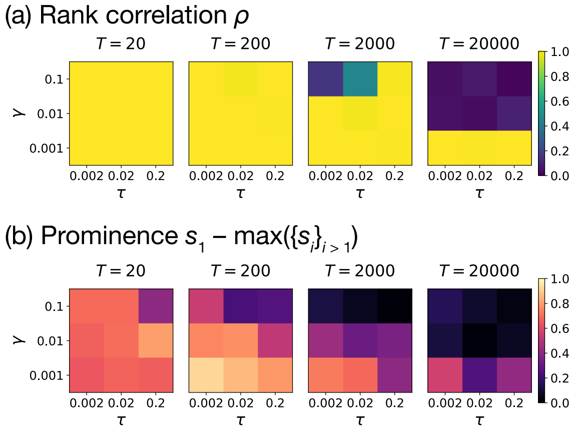

Supplementary Figure 5 Effect of varying γ and τ on the identification of an approximate conservation law for the Langevin harmonic oscillator. Varying γ, τ, and the sampling time T, we show (a) the rank correlation ρ of the first embedding component with the time-averaged energy $\langle E \rangle$ and (b) the prominence $s_1 - \max(\{s_i\}_{i>1})$ of the score of the first component s_1 over the highest score of the remaining components s_i.

Supplementary Note 4 Nonlinear Periodic Orbit of the Double Pendulum

In addition to the chaotic and linear non-chaotic phases, the double pendulum can also exhibit other kinds of complex behavior, including highly nonlinear periodic orbits. In our extracted embedding (Fig. 6a), we see an example of such a nonlinear periodic orbit (Figs. 6b,c). The placement of this periodic orbit in the embedding also meaningfully connects it with the low energy in-phase mode from the linear coupled oscillator regime (Fig. 5g), i.e. this periodic orbit can be thought of as a nonlinear high energy extension of the low energy in-phase mode.

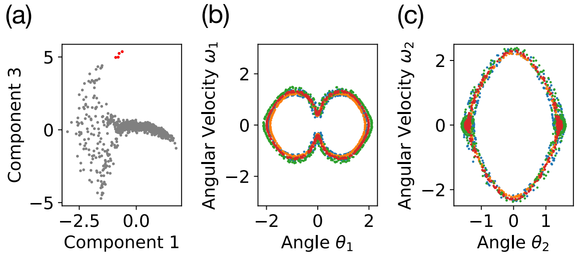

Supplementary Figure 6 Nonlinear periodic orbit of the double pendulum. (a) The four red highlighted points in the extracted embedding correspond to (b, c) a periodic orbit of the double pendulum that is connected to but well outside of the linear coupled oscillator regime.

Supplementary Note 5 Robustness to Noise & Partial Observations

To further demonstrate the robustness of our approach, we show several additional experiments on the simple pendulum, planar gravitational dynamics, and double pendulum datasets. For the simple pendulum, our method still performs well when using only angle θ measurements, i.e. a partially observed phase space (Fig. 7a). In fact, even if we add Gaussian noise (standard deviation $\sigma = 0.5$) to the raw trajectory data in addition to using a partially observed phase space, we still obtain a similar result (Fig. 7b). Similarly, for planar gravitational dynamics with only position $\mathbf{r}$ data or with added Gaussian noise ($\sigma = 0.5$), our method is still able to identify the three conserved quantities (Figs. 7c,d). For the double pendulum, we again see that we retain the same detail in the extracted embedding using only position data (Fig. 8). The corresponding rank correlations with the ground truth conserved quantities are given in Table 1.

The robustness of our method to both noise and partial observations is largely a consequence of using the Wasserstein distance as our metric for comparing trajectories. In our problem formulation, we consider the isosurfaces with constant conserved quantities and ask for a metric for measuring distances between isosurfaces. Because the Wasserstein distance measures distances between distributions rather than disjoint isosurfaces, it can easily generalize to noisy or partially observed data where the trajectories are no longer strictly disjoint. An alternative choice of metric, e.g. the Hausdorff distance between sets in a metric space [S10], does not have this nice property and would be much more susceptible to noise or partial observations. On the other hand, because the Wasserstein distance distinguishes between different distributions, it is susceptible to other forms of corruption such as sampling inhomogeneity (discussed in Supplementary Note 7.3). In most cases, we believe that this tradeoff is worth it to retain the high degree of robustness as long as one is aware of the limitations.

Supplementary Table 1 Rank correlations ρ of linear fits with ground truth conserved quantities for the additional experiments. *The rank correlations for the low energy approximately conserved mode energies $E_\pm$ are computed on the restricted set of trajectories with first embedding component $v_1 < -1$.

Dataset	Conserved Quantity	ρ
Simple Pendulum: *Position Only*	E	0.998
Simple Pendulum: *Position Only + Noise*	E	0.996
Planar Gravitational Dynamics: *Position Only*	$a \cos \phi$<br>$a \sin \phi$<br>L	0.994<br>0.993<br>0.968
Planar Gravitational Dynamics: *Noise*	$a \cos \phi$<br>$a \sin \phi$<br>L	0.994<br>0.992<br>0.945
Double Pendulum: *Position Only*	E<br>E_+<br>E_-	0.996<br>0.931*<br>0.945*

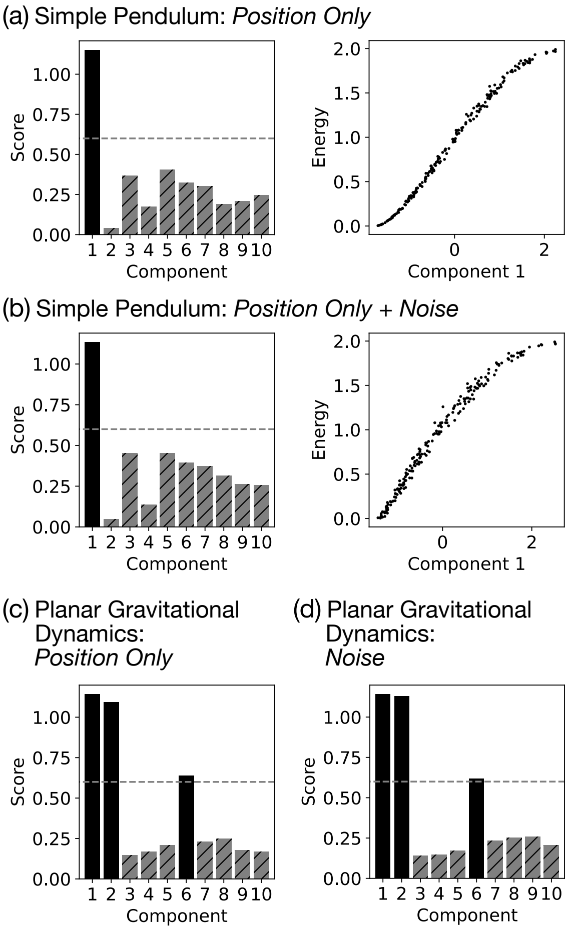

Supplementary Figure 7 Additional experiments illustrating the robustness of our approach. (a) For the simple pendulum system, even when provided only angle θ measurement data (without angular velocity ω), our method is able to identify a single relevant component corresponding to the energy the pendulum ($\rho = 0.998$). (b) If we then also add $\sigma = 0.5$ Gaussian noise, we can still achieve a similar result ($\rho = 0.996$). For planar gravitational dynamics, our method also performs well given (c) only position $\mathbf{r}$ data or (d) with $\sigma = 0.5$ Gaussian noise, correctly identifying the three conserved quantities.

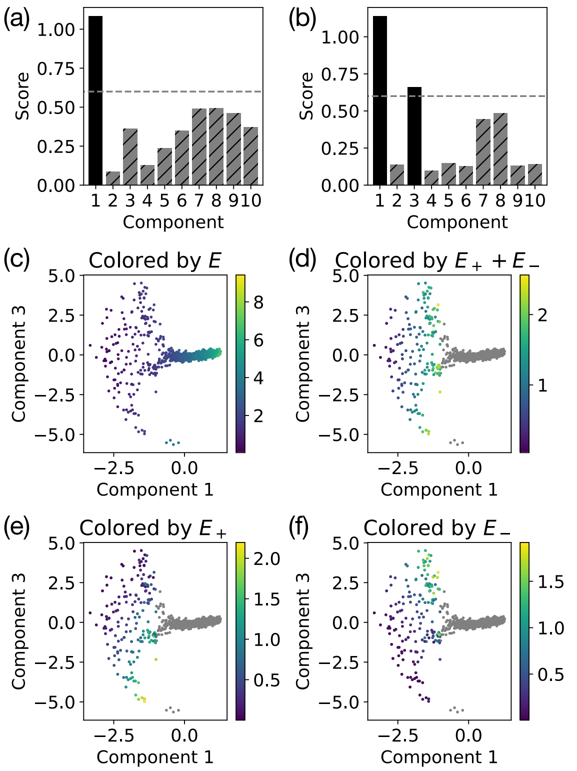

Supplementary Figure 8 Identifying conserved quantities for the double pendulum from only position data. (a) The heuristic score (with cutoff 0.6) identifies one relevant embedding component corresponding to (b) the total energy E. (c) However, if we restrict the embedding to trajectories with first component $v_1 < -1$ (i.e. low energy trajectories) and renormalize the embedding, we find (d–f) two conserved quantities corresponding to the energies $E_\pm$ of the two decoupled low energy modes. The gray points in Figures 8d–f correspond to the high energy trajectories (first component $v_1 > -1$) which are not relevant when considering the low energy non-chaotic phase of the double pendulum.

Supplementary Note 6 Comparison with Direct Fitting Methods

Only a few alternative approaches [14–16] have been proposed for identifying conservation laws from trajectory samples without time information (e.g. any methods that require time derivative estimates of the system state are not applicable). These alternatives all fall into a broad category that we term "direct fitting" methods, which attempt to directly fit a parameterized function of the system state to be constant over each trajectory. This can be accomplished using a variety of methods and optimization objectives but all generally require that the system state is fully observed and that the observed trajectories have low noise. These restrictions ensure that the basic assumption of direct fitting methods—that there exists a well-defined function from the observed state to the conserved quantities—is fulfilled.

Supplementary Table 2 Performance comparison of our method alongside deep learning-based direct fitting methods on the planar gravitational dynamics dataset. We show the rank correlation ρ of the embeddings with the ground truth conserved quantities. Note that the directing fitting approaches, ConservNet and Siamese Neural Network (SNN), are designed to discover a single conserved quantity and cannot handle multiple conserved quantities without dataset regeneration. Thus, with only a single dataset, we only expect the direct fitting methods to learn a single conserved quantity. For the direct fitting methods, when fitting to conserved quantities involving the orientation angle ϕ, we choose a constant ϕ_0 that gives the maximum ρ with conserved quantities of the form $a\cos(\phi - \phi_0)$. Bolded numbers indicate the best rank correlations for each conserved quantity.

Dataset	Method	Conserved Quantity	ρ
Clean, Fully Observed	Manifold Learning (Ours)	$a\cos\phi$	**0.994**
		$a\sin\phi$	**0.992**
		L	**0.970**
	ConservNet	$a\cos(\phi - \phi_0)$	0.882
		$a\sin(\phi - \phi_0)$	0.029
		L	0.002
	SNN	$a\cos(\phi - \phi_0)$	0.948
		$a\sin(\phi - \phi_0)$	0.024
		L	0.007
Noisy ($\sigma = 0.5$), Fully Observed	Manifold Learning (Ours)	$a\cos\phi$	**0.994**
		$a\sin\phi$	**0.992**
		L	**0.945**
	ConservNet	$a\cos(\phi - \phi_0)$	0.178
		$a\sin(\phi - \phi_0)$	0.003
		L	0.069
	SNN	$a\cos(\phi - \phi_0)$	0.031
		$a\sin(\phi - \phi_0)$	0.005
		L	0.025
Clean, Partially Observed (Position Only)	Manifold Learning (Ours)	$a\cos\phi$	**0.994**
		$a\sin\phi$	**0.993**
		L	**0.968**
	ConservNet	$a\cos(\phi - \phi_0)$	0.892
		$a\sin(\phi - \phi_0)$	0.036
		L	0.015
	SNN	$a\cos(\phi - \phi_0)$	0.916
		$a\sin(\phi - \phi_0)$	0.010
		L	0.060

AI Poincaré [14] uses a symbolic regression approach to directly obtain an interpretable expression for the conserved quantities in terms of a library of symbolic expressions. While this method can sometimes give the exact expected conservation laws, AI Poincaré relies heavily on the applicability of symbolic regression, and, in cases where there is no simple symbolic expression, it will often fail. For example, for the planar gravitational dynamics dataset (named the "Kepler problem" in [14]), AI Poincaré fails to identify the conserved quantity associated to the orientation angle of the orbit due to its somewhat awkward symbolic representation [14].

The two remaining direct fitting methods, Siamese Neural Network (SNN) [15] and ConservNet [16], both use a neural network as the parameterized function to fit the trajectory data. One important limitation of both of these approaches is their inability to discover more than a single conserved quantity per dataset. In both works [15, 16], identifying a second conserved quantity requires generating a new dataset while holding the first discovered conserved quantity fixed. Generating such a dataset as part of the method pipeline assumes both access to and fine-grained control of the data generating process, which is often not available in practice. We benchmark our manifold learning approach against these two methods on the planar gravitational dynamics dataset, variations of which (under the name "Motion in a central potential" and "Kepler problem") were also previously studied in both works [15, 16]. Because we only use a single dataset, we only expect these direct fitting methods to identify a single conserved quantity. We also compare these methods on the noisy and partially observed versions of the dataset to understand their limitations.

Implementations for both direct fitting methods were adapted from code released by [16] since reference code for [15] is unavailable. Following [16], our benchmark uses a simple fully connected neural network with four hidden layers of size 320, Mish activations [S11], and an Adam optimizer [S12] with learning rate 5×10^{-5}. We use a batch size of 1000 for the SNN and 200 (fixed by the number of samples per trajectory) for ConservNet. We train each network for 1000 epochs and then compute the rank correlation ρ of the fitted function with the ground truth conserved quantities (Table 2). We did not see any improvement on our dataset when the networks were trained for 50,000 epochs, as recommended by [16].

The results show that, as expected, the direct fitting methods only learned a single conserved quantity for the dataset (Table 2). Even for the single conserved quantity identified by SNN and ConservNet, we see that our manifold learning method outperforms both direct fitting methods in all settings while also identifying all three conserved quantities. Training for SNN and ConservNet took between 40–50 minutes for 1000 epochs on a single RTX 2080 Ti GPU and does not appear to benefit significantly from using larger batch sizes and additional compute resources. In comparison, our method—which is limited primarily by the Wasserstein distance estimation (Supplementary Note 7.1)—takes around 40 seconds to run on eight RTX 2080 Ti GPUs and 5–6 minutes on a single RTX 2080 Ti GPU.

Supplementary Note 7 Additional Discussion & Limitations

7.1 Time & Space Complexity

For a dataset with N trajectories and with S samples per trajectory, our approach is currently limited by the computational cost of estimating the 2-Wasserstein distance for all pairs of trajectories (Table 3), giving a time complexity of $\mathcal{O}(N^2 S^2 \log S/\varepsilon^2)$ (Supplementary Note 2.1). To scale to much larger datasets, we have several choices to improve the run time performance.

Supplementary Table 3 Run times for computing the pairwise Wasserstein distances for each dataset. In addition to the run times, we also list the number of trajectories N in the dataset, the number of samples S per trajectory, and the dimension d of the phase space. We used eight RTX 2080 Ti GPUs for all computations. †Because we are interested in local conservation laws for the KdV equation dataset, the phase space is treated differently (see Sec. 2.2.6).

Dataset	N	S	d	Run Time
SHO	200	200	2	11 s
Simple Pendulum	200	200	2	11 s
Planar Grav. Dynamics	400	200	4	40 s
Double Pendulum	1000	500	4	54 m 43 s
Osc. Turing Patterns	400	200	100	54 s
KdV Equation	400	200	$200^{\dagger}$	26 m 16 s

One simple adjustment to speed up the convergence of the Sinkhorn algorithm is to allow for a significantly larger target regularization parameter γ. The result, in fact, interpolates between the Wasserstein metric ($\gamma = 0$) and a maximum mean discrepancy (MMD) metric ($\gamma = \infty$) [29]. However, to improve the time complexity of our approach as we scale to large S, we may have to consider more approximate approaches. A popular option is to first subsample the data to form minibatches of size $s \ll S$, solve the much smaller optimal transport problem, and then average the resulting estimates for the Wasserstein distance [S13, S14]. For a fixed number of epochs of averaging, this approach would give us linear scaling $\mathcal{O}(S)$ with the number of samples S.

To improve the scaling with the number of trajectories N, we may be able to take advantage of the sparse structure of the kernel matrix K when the total number of conserved quantities $n \ll N$. That is, when the dimension of the embedded manifold (corresponding to the conserved quantities) is low, we expect each trajectory to have relatively few nearest neighbors, so most entries of the kernel matrix K will be very close to zero for an appropriately chosen Gaussian kernel. If that is the case, we can construct the kernel matrix K as a sparse matrix, e.g. by starting with a very coarse approximation for the pairwise Wasserstein distances and then obtaining finer estimates only for nearby trajectories or by constructing a k-nearest neighbor tree [S15] (which takes $\mathcal{O}(kN \log N)$ time). With a sparse kernel matrix K, the diffusion map will only take $\mathcal{O}(N)$ time and use $\mathcal{O}(N)$ space.

In the future, by incorporating these approximations and algorithmic improvements, we expect to be able to adapt our approach to achieve linear scaling in time and space for very large datasets.

7.2 Sample Complexity

To understand the sample complexity of our approach, we need to consider the effect of both the number of trajectories N and the number of samples per trajectory S.

The number of samples S determines the accuracy of the estimated pairwise Wasserstein distances. For the Sinkhorn algorithm, the approximation error is [S16]

$$\mathcal{O}\big(S^{-1/2}\, e^{\kappa/\gamma}\big(1 + \gamma^{-\lfloor d/2 \rfloor}\big)\big), \tag{S11}$$

where d is the dimension of the data, γ is the entropy regularization parameter, and κ is a data-dependent constant. That is, the error in our Wasserstein distance estimates scales as $1/\sqrt{S}$. Also, for $\gamma \geq 1$, notice that the dimension d has no significant influence on the error bound. Furthermore, the relevant dimension d in the bound is likely to be some measure of the intrinsic dimension of the data [S17], allowing us to obtain good Wasserstein distance estimates even for some systems with high dimensional phase spaces (Sec. 2.2.5).

Assuming we have accurate Wasserstein distance estimates, the spectral error of the diffusion map is $\mathcal{O}(N^{-2/(8+n)})$ for a manifold of dimension n (i.e. the number of conserved quantities) [S18]. Thus, for integrable PDE systems like the KdV equation (Sec. 2.2.6) with an infinite number of conserved quantities, we must restrict ourselves to considering local conserved quantities to have a reasonable chance of reconstructing a useful embedding.

7.3 Robustness & Sampling Inhomogeneity

Because the Wasserstein distance is a metric over distributions, it has great robustness properties (Supplementary Note 5). However, the exact same qualities that provide this robustness also make the Wasserstein metric sensitive to sampling inhomogeneity between trajectories. That is, if two trajectories with the same conserved quantities are sampled in a way such that their distributions over the measured phase space differ, then the Wasserstein metric will treat them as different distributions, which could lead to spurious conserved quantities being identified by our method. Note that this does not include changes in the sampling process that are uniform across the trajectories and only a function of the phase space, e.g. sampling the state of the pendulum more often when it is near to the bottom of its swing, or sampling the position of a planet more often when it is farther from the sun for all trajectories.

One example of the relevant kind of inhomogeneity is sampling trajectories for too short a time such that the physical measure (Eq. 18) is not well approximated. Trajectories sampled over too short a time can essentially lead our method to believe that there are additional conservation laws preventing the system state from exploring areas of phase space that may in fact be reachable for a longer trajectory. On the hand, this can be considered a feature rather than a bug in the sense that, by choosing the time over which to sample our trajectories, we are providing our method with a time scale for our conserved quantities, allowing us to probe approximately conserved quantities that appear invariant over shorter time scales (see Supplementary Note 3 for an example).

If we have a fully observed phase space and low noise, then this effect can be mitigated by choosing an alternative metric, such as a Hausdorff distance [S10], that does not have a strong dependence on the sampling distribution. This would essentially take advantage of the fact that any overlap between two trajectories in a fully observed phase space implies that both have the same conserved quantities. However, for noisy data or a partially observed phase space, observed overlap between measured trajectories could also be due to noise or hidden variables. Again, the same qualities that make the Hausdorff distance robust to sampling inhomogeneity also make it very sensitive to noise and partial observations.

In other words, the Wasserstein metric is able to distinguish trajectories with different conserved quantities even in a noisy partially observed phase space precisely because it can sense differences in the distributions of the trajectories.

7.4 Physical Measures, Dissipation, & Unbounded Dynamics

The assumption that the dynamical system admits a physical measure (Sec. 4.1.2) is a fairly natural one that allows us to characterize conserved quantities using invariant measures. In fact, for Hamiltonian dynamics that already admit a canonical Liouville measure [S19], assuming that trajectories are ergodic

on the isosurfaces of conserved quantities is essentially Boltzmann's famous ergodic hypothesis [S20, S21], which underpins much of statistical mechanics but is notoriously difficult to prove in general.

For dissipative systems, our assumption as stated is false since dissipation generally destroys ergodicity. However, dissipative systems still have attractors that admit physical measures [31][S22]. Therefore, for a dissipative system, rather than characterizing ergodic measures on isosurfaces, our method would instead be characterizing the various attractors of the dynamics, including fixed points, limit cycles, and chaotic attractors. Our experiment with the oscillating Turing pattern (Sec. 2.2.5) is precisely such a dissipative system with a continuous set of chaotic attractors parameterized by a spatial phase angle η.

One clear example where our assumption of a physical measure is violated in a meaningful way is when the dynamics are unbounded. For example, hyperbolic orbits following planar gravitation dynamics escape to infinity and do not converge to any invariant measure. In this case, naively applying our approach would not lead to meaningful results since, for any fixed sampling time, two different trajectories from the same hyperbolic orbit will sample very different portions of phase space depending on initial conditions. One potential solution for this issue of unbounded dynamics is to first compactify the phase space.

For example, if we compactify the traditional four dimensional Euclidean phase space of planar gravitational dynamics into a four dimensional real projective space with an attached metric (e.g. the Fubini-Study metric) [S23], we have effectively made the dynamics bounded and therefore amenable to our approach. Moving to this projective representation, the physical measures on the elliptical orbits would remain largely unchanged while trajectories on hyperbolic orbits would approach a fixed point at the "line at infinity" (which would be a finite "distance" away due to the choice of metric on this compactified space). Our method would then be able to characterize the elliptical orbits by the usual three conserved quantities and hyperbolic orbits by their limiting long time behavior corresponding to their final velocity vectors as they escape to infinity.

7.5 Trajectory Diversity & Correlated Conserved Quantities

One fundamental limitation, which affects not only our method but also other approaches for discovering conservation laws [12–16], is the inability to distinguish between constraints and correlated conserved quantities. In general, constraints on the system dynamics are constant across all possible initial conditions, whereas conserved quantities vary based on initial condition. However, if there is a lack of diversity in the initial conditions for the trajectories in our dataset, then it is possible to mistake two highly correlated conserved quantities for an additional constraint on the system. For example, if the planar gravitational dynamics dataset only contained nearly circular orbits, then the angular momentum and the energy of the orbits will become highly correlated and the orientation angle will be essentially meaningless. Given such a dataset of circular trajectories and no additional information, it is not possible to distinguish between a planar gravitational dynamics dataset with poor trajectory diversity and a dataset from a system that is constrained to circular orbits with a single conserved quantity (associated with the radius of the orbits). As such, any general method for discovering conserved quantities will treat two highly correlated conserved quantities as a single conserved quantity.

Supplementary Note 8 Dataset Details

The SHO dataset contains 200 sample trajectories, each with 200 uniformly sampled states in time.

The simple pendulum dataset contains 200 trajectories with uniformly sampled energies $E \in [0, 2]$. Each trajectory has 200 sampled states at uniformly sampled times $t \in [0, 2000]$.

The planar gravitational dynamics dataset contains 400 trajectories with uniformly sampled energies $E \in [-0.15, -0.5]$, angular momenta $L \in [0, 1]$, and orbital orientation angles $\phi \in [0, 2\pi)$. Each trajectory has 200 sampled states at uniformly sampled times $t \in [0, 2000]$.

The double pendulum dataset contains 1000 trajectories with initial angles $\theta_1, \theta_2 \sim \text{Unif}(-0.75\pi, 0.75\pi)$ and initial angular velocities $\omega_1, \omega_2 \sim N(0, 0.5^2)$. Each trajectory contains 500 points uniformly sampled in time $t \in [0, 50000]$. One additional subtlety of applying our approach to the double pendulum comes from the periodicity of the angles θ_1, θ_2 describing the positions of the two pendulums. The Euclidean ground metric used for optimal transport must take into account this periodicity, so we choose to leave the data unnormalized and use the shortest Euclidean distance between pairs of points in the periodic phase space.

The oscillating Turing pattern dataset contains 400 trajectories, where we initialize our states $u(x)$ and $v(x)$ with unit Gaussian noise in Fourier space and take 200 states with uniformly sampled times $t \in [300, 1300]$. By allowing for a transient time of 300, we focus our study on the long term behavior of the oscillating Turing pattern.

Finally, we study the KdV equation on a periodic domain of size $l = 20$ and with mesh size 200 (downsampled from a mesh size of 1000 used during data generation). The dataset contains 400 trajectories each with 200 states at uniformly sampled times $t \in [0, 10]$. To produce a reasonable variety of initial conditions, each trajectory is initialized with normally distributed Fourier components scaled by a Gaussian band-limiting envelope with width uniformly sampled in the interval $[10\pi/l, 20\pi/l]$.

Supplementary Note 9 Proof of Optimal Transport for the Simple Harmonic Oscillator

Let the transport cost between a pair of points $(\theta_i, \theta_j) \in S^1 \times S^1$ be

$$c(\theta_i, \theta_j) = k_q^2(r_1 \cos\theta_i - r_2 \cos\theta_j)^2 + k_p^2(r_1 \sin\theta_i - r_2 \sin\theta_j)^2. \tag{S12}$$

Then, for the proposed optimal transport plan Π with support Γ containing all points $(\theta, \theta) \in S^1 \times S^1$, we will show that Γ is c-cyclically monotone, and therefore Π is optimal. See Medio and Lines [31] for further details.

To demonstrate this fact, consider a finite set of pairs $\{(\theta_1, \theta_1), (\theta_2, \theta_2), \dots, (\theta_n, \theta_n)\} \subset \Gamma$. Restricted to this finite set, the total cost given the transport plan Π is

$$C = \frac{1}{n}\sum_{i=1}^{n} c(\theta_i, \theta_i) \tag{S13}$$

$$= \frac{r_1^2 + r_2^2}{n}\sum_{i=1}^{n}(k_q^2 \cos^2\theta_i + k_p^2 \sin^2\theta_i) - \frac{2r_1r_2}{n}\sum_{i=1}^{n}(k_q^2 \cos^2\theta_i + k_p^2 \sin^2\theta_i). \tag{S14}$$

Now, consider an alternative transport plan Π' with support $\{(\theta_1, \theta_2), (\theta_2, \theta_3), \dots, (\theta_n, \theta_1)\}$ forming a cycle. The total cost is given by

$$C' = \frac{1}{n}\sum_{i=1}^{n} c(\theta_i, \theta_{i+1}) \tag{S15}$$

$$= \frac{r_1^2 + r_2^2}{n}\sum_{i=1}^{n}(k_q^2 \cos^2\theta_i + k_p^2 \sin^2\theta_i) - \frac{2r_1r_2}{n}\sum_{i=1}^{n}(k_q^2 \cos\theta_i \cos\theta_{i+1} + k_p^2 \sin\theta_i \sin\theta_{i+1}), \tag{S16}$$

where we let $\theta_{n+1} = \theta_1$. Then, the difference

$$\begin{aligned} C' - C = {} & \frac{2r_1r_2k_q^2}{n}\sum_{i=1}^{n}\left[\frac{\cos^2\theta_i + \cos^2\theta_{i+1}}{2} - \cos\theta_i \cos\theta_{i+1}\right] \\ & + \frac{2r_1r_2k_p^2}{n}\sum_{i=1}^{n}\left[\frac{\sin^2\theta_i + \sin^2\theta_{i+1}}{2} - \sin\theta_i \sin\theta_{i+1}\right] \\ \geq {} & 0, \end{aligned} \tag{S17}$$

since

$$\frac{\cos^2\theta_i + \cos^2\theta_{i+1}}{2} \geq \cos\theta_i \cos\theta_{i+1} \tag{S18}$$

and

$$\frac{\sin^2\theta_i + \sin^2\theta_{i+1}}{2} \geq \sin\theta_i \sin\theta_{i+1} \tag{S19}$$

by the AM–GM inequality (and is trivially true if the right hand side is negative). Therefore, any such cycle will result in an equal or higher transport cost (strictly higher if at least one pair θ_i, θ_{i+1} are distinct), implying that Γ is c-cyclically monotone.

Supplementary References

[S1] Altschuler, J., Niles-Weed, J., Rigollet, P.: Near-linear time approximation algorithms for optimal transport via sinkhorn iteration. In: Guyon, I., Luxburg, U.V., Bengio, S., Wallach, H., Fergus, R., Vishwanathan, S., Garnett, R. (eds.) Advances in Neural Information Processing Systems, vol. 30. Curran Associates, Inc., Long Beach, CA, USA (2017). https://proceedings.neurips.cc/paper/2017/file/491442df5f88c6aa018e86dac21d3606-Paper.pdf

[S2] Dvurechensky, P., Gasnikov, A., Kroshnin, A.: Computational optimal transport: Complexity by accelerated gradient descent is better than by sinkhorn's algorithm. In: Dy, J., Krause, A. (eds.) Proceedings of the 35th International Conference on Machine Learning. Proceedings of Machine Learning Research, vol. 80, pp. 1367–1376. PMLR, Stockholm, Sweden (2018). https://proceedings.mlr.press/v80/dvurechensky18a.html

[S3] Cuturi, M., Meng-Papaxanthos, L., Tian, Y., Bunne, C., Davis, G., Teboul, O.: Optimal Transport Tools (OTT): A JAX Toolbox for all things Wasserstein. arXiv (2022). https://doi.org/10.48550/ARXIV.2201.12324

[S4] Roweis, S.T., Saul, L.K.: Nonlinear dimensionality reduction by locally linear embedding. Science **290**(5500), 2323–2326 (2000). https://doi.org/10.1126/science.290.5500.2323

[S5] Zhang, Z., Zha, H.: Principal manifolds and nonlinear dimensionality reduction via tangent space alignment. SIAM Journal on Scientific Computing **26**(1), 313–338 (2004). https://doi.org/10.1137/S1064827502419154

[S6] Tenenbaum, J.B., de Silva, V., Langford, J.C.: A global geometric framework for nonlinear dimensionality reduction. Science **290**(5500), 2319–2323 (2000). https://doi.org/10.1126/science.290.5500.2319

[S7] Karoui, N.E., Wu, H.-T.: Graph connection Laplacian methods can be made robust to noise. The Annals of Statistics **44**(1), 346–372 (2016). https://doi.org/10.1214/14-AOS1275

[S8] Bengio, Y., Paiement, J.-f., Vincent, P., Delalleau, O., Roux, N., Ouimet, M.: Out-of-sample extensions for lle, isomap, mds, eigenmaps, and spectral clustering. In: Thrun, S., Saul, L., Schölkopf, B. (eds.) Advances in Neural Information Processing Systems, vol. 16. MIT Press, Vancouver, British Columbia, Canada (2003). https://proceedings.neurips.cc/paper/2003/file/cf05968255451bdefe3c5bc64d550517-Paper.pdf

[S9] Long, A.W., Ferguson, A.L.: Landmark diffusion maps (l-dmaps): Accelerated manifold learning out-of-sample extension. Applied and Computational Harmonic Analysis **47**(1), 190–211 (2019). https://doi.org/10.1016/j.acha.2017.08.004

[S10] Feydy, J., Trouvé, A.: Global divergences between measures: From hausdorff distance to optimal transport. In: Reuter, M., Wachinger, C., Lombaert, H., Paniagua, B., Lüthi, M., Egger, B. (eds.) Shape in Medical Imaging, pp. 102–115. Springer, Cham (2018)

[S11] Misra, D.: Mish: A Self Regularized Non-Monotonic Activation Function. arXiv (2019). https://doi.org/10.48550/ARXIV.1908.08681

[S12] Kingma, D.P., Ba, J.: Adam: A Method for Stochastic Optimization. In: International Conference on Learning Representations (2015)

[S13] Genevay, A., Peyre, G., Cuturi, M.: Learning generative models with sinkhorn divergences. In:

Storkey, A., Perez-Cruz, F. (eds.) Proceedings of the Twenty-First International Conference on Artificial Intelligence and Statistics. Proceedings of Machine Learning Research, vol. 84, pp. 1608–1617. PMLR, Lanzarote, Spain (2018). https://proceedings.mlr.press/v84/genevay18a.html

[S14] Fatras, K., Zine, Y., Flamary, R., Gribonval, R., Courty, N.: Learning with minibatch wasserstein: asymptotic and gradient properties. In: Chiappa, S., Calandra, R. (eds.) Proceedings of the Twenty Third International Conference on Artificial Intelligence and Statistics. Proceedings of Machine Learning Research, vol. 108, pp. 2131–2141. PMLR, Palermo, Italy (2020). https://proceedings.mlr.press/v108/fatras20a.html

[S15] Friedman, J.H., Bentley, J.L., Finkel, R.A.: An algorithm for finding best matches in logarithmic expected time. ACM Trans. Math. Softw. **3**(3), 209–226 (1977). https://doi.org/10.1145/355744.355745

[S16] Genevay, A., Chizat, L., Bach, F., Cuturi, M., Peyré, G.: Sample complexity of sinkhorn divergences. In: Chaudhuri, K., Sugiyama, M. (eds.) Proceedings of the Twenty-Second International Conference on Artificial Intelligence and Statistics. Proceedings of Machine Learning Research, vol. 89, pp. 1574–1583. PMLR, Naha, Okinawa, Japan (2019). https://proceedings.mlr.press/v89/genevay19a.html

[S17] Weed, J., Bach, F.: Sharp asymptotic and finite-sample rates of convergence of empirical measures in Wasserstein distance. Bernoulli **25**(4A), 2620–2648 (2019). https://doi.org/10.3150/18-BEJ1065

[S18] Wormell, C.L., Reich, S.: Spectral convergence of diffusion maps: Improved error bounds and an alternative normalization. SIAM Journal on Numerical Analysis **59**(3), 1687–1734 (2021). https://doi.org/10.1137/20M1344093

[S19] Goldstein, H., Poole, C.P., Safko, J.L.: Classical Mechanics. Addison Wesley, Boston, MA, USA (2002)

[S20] Pathria, R.K., Beale, P.D.: Statistical Mechanics. Elsevier Science, London, UK (2021)

[S21] Walters, P.: An Introduction to Ergodic Theory. Graduate Texts in Mathematics. Springer, New York, NY, USA (2000)

[S22] Palis, J.: A global perspective for non-conservative dynamics. Annales de l'Institut Henri Poincaré C, Analyse non linéaire **22**(4), 485–507 (2005). https://doi.org/10.1016/j.anihpc.2005.01.001

[S23] Tu, L.W.: An Introduction to Manifolds. Universitext. Springer, New York, NY, USA (2007)
